# Decoupling of Spin and Non‐Spin Effects From Electronic Structure Modulation for Oxygen Electrocatalysis

**DOI:** 10.1002/advs.202514540

**Published:** 2025-11-08

**Authors:** Yingliang Zhao, Zhen Ji, Xiang Xiao, Zhi Fang, Yanglong Hou

**Affiliations:** ^1^ School of Materials Shenzhen Campus of Sun Yat‐sen University Guangdong 518107 China; ^2^ School of Materials Science and Engineering Peking University Beijing 100871 China

**Keywords:** deep decoupling, electronic structure, oxygen‐involved reactions, spin and non‐spin effects, spin‐dependent electrocatalysis

## Abstract

The aggravation of global warming and environmental degradation amplifies the demand for green energy technologies, where oxygen‐involved reactions, including oxygen evolution reaction (OER) and oxygen reduction reaction (ORR), play pivotal roles in energy conversion. However, they suffer from inherent spin‐related kinetic bottlenecks due to the spin transitions between singlet H_2_O and triplet O_2_. While the modulation strategies (built‐in electric fields, built‐in magnetic fields, crystal fields and ligand fields) advance electronic structure engineering for catalyst design. Nevertheless, they inevitably co‐regulate spin effects (spin state/polarization) and non‐spin effects (d‐band center shifts, valence tuning, etc.), making it difficult to distinguish the individual contributions to catalytic performance. This review systematically distinguishes the influence of spin/non‐spin factors in electronic structure‐modulated OER/ORR, aiming to clarify their regulatory mechanisms, contribution ratios, and synergistic effects. Statistical analyses indicate that spin‐related and non‐spin‐related effects exhibit comparable importance, with synergistic regulation yielding superior OER performance. Further analyzing their distinct roles in dynamics (adsorption/desorption) and kinetics (spin flip/selective electron transfer), this work proposes a deeply decoupling approach to disentangle these effects, providing new perspectives for rational design of spin‐controlled electrocatalysts and optimizing catalytic performance beyond traditional constraints.

## Graphical abstract

The strategies of electronic structure modulation in spin catalysis inevitably induce both spin and non‐spin effects, making it challenging to isolate their individual contributions to catalytic performance. To clarify dominant factors, spin/non‐spin effects from electronic structure regulation require systematic decoupling (i.e., through constructing comparable reference experiments, DFT calculations and mathematical analysis) and precise spin characterizations (e.g., in situ XES, XAS, XMCD).

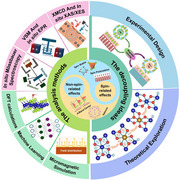

## 1 Introduction

The intensification of global warming and environmental degradation has significantly heightened the urgent demand for green and clean energy, as well as novel energy conversion materials.^[^ ^1^, ^2^, ^3^, ^4^, ^5^, ^6^, ^7^ ^]^ In this context, technologies such as water electrolysis for hydrogen production, rechargeable metal‐air batteries, and fuel cells play pivotal roles in energy conversion and storage.^[^ ^8^, ^9^, ^10^, ^11^, ^12^, ^13^, ^14^, ^15^ ^]^ The electrocatalytic process, as the core link of these technologies, directly determines energy conversion efficiency. In electrochemical water splitting for hydrogen production, the kinetically sluggish step is the oxygen evolution reaction (OER).^[^ ^16^, ^17^, ^18^ ^]^ For fuel cells, the key bottleneck limiting their breakthrough lies in the oxygen reduction reaction (ORR).^[^ ^19^, ^20^, ^21^ ^]^ In contrast, rechargeable metal‐air batteries require efficient performance for both OER and ORR, thus necessitating excellent bifunctional catalysts.^[^ ^22^, ^23^, ^24^, ^25^ ^]^ However, the key oxygen‐involved reactions (OER and ORR) involve four‐electron transfer processes, and their kinetic sluggishness severely restricts electrocatalytic efficiency and the practical application of related technologies.^[^ ^4^, ^26^, ^27^, ^28^, ^29^ ^]^ Consequently, the development of efficient oxygen‐involved electrocatalysis has become a critical task for researchers.

Currently, high‐efficiency electrocatalysts remain heavily reliant on noble metals (e.g., Pt, Ru, Rh, Pd, Ir) and their oxides (e.g., IrO_2_, RuO_2_).^[^ ^30^, ^31^, ^32^ ^]^ Yet, their exorbitant cost and resource scarcity severely restrict large‐scale implementation, making the development of low‐cost, novel oxygen‐involved catalysts a key challenge. Over the past two decades, compared with traditional empirical trial‐and‐error methods, the strategies of electronic structure modulation (including built‐in electric fields,^[^ ^33^, ^34^ ^]^ built‐in magnetic fields,^[^ ^35^, ^36^ ^]^ crystal fields,^[^ ^37^, ^38^ ^]^ and ligand fields.^[^ ^39^, ^40^ ^]^) have made significant progress. These strategies not only demonstrate excellent catalytic performance optimization but also provide theoretical guidance for the rational design of high‐performance catalysts through mechanistic studies.

Recently, the major experimental approaches, such as doping,^[^ ^41^, ^42^ ^]^ stress‐strain engineering,^[^ ^43^, ^44^, ^45^ ^]^ defect/vacancy introduction,^[^ ^46^, ^47^ ^]^ heterostructure construction,^[^ ^48^, ^49^ ^]^ surface reconstruction,^[^ ^50^, ^51^, ^52^ ^]^ coordination modification,^[^ ^53^, ^54^ ^]^ intrinsically modulate the electronic structure of catalysts, including built‐in electric fields, built‐in magnetic fields, crystal fields, and coordination fields. They serve as core methods for tuning material properties and electrocatalysts performance.

Notably, the four‐electron transfer process of oxygen‐involved reactions inherently involves spin‐dependent electron transitions between singlet H_2_O and triplet O_2_.^[^ ^7^, ^14^, ^55^, ^56^ ^]^ The reaction necessitates spin polarization to facilitate the parallel alignment of two electrons, thereby assisting the conversion of triplet oxygen. Recent theoretical calculations and experimental studies have revealed that spin properties exert non‐negligible influences on the thermodynamic and kinetic processes of oxygen‐involved reactions, with a spin‐flip energy barrier of ≈1.1 eV needing to be surmounted. By modulating the spin electronic structure of catalysts, this spin‐flip energy barrier can be effectively eliminated, fundamentally breaking through spin‐dependent kinetic limitations and establishing as a new paradigm beyond traditional thermodynamic/kinetic constraints.^[^ ^7^, ^31^, ^57^, ^58^ ^]^ Nevertheless, while regulating spin‐related effects (e.g., spin states and spin polarization), above experimental approaches inevitably induce non‐spin‐related effects, including d‐band center shifts, valence tuning, conductivity changes, and so on. It is a major challenge to distinguish the contribution of spin‐related and non‐spin‐related effects on catalytic performance, which makes the mechanism of spin catalysis remain unclear.

Meanwhile, current research has primarily focused on non‐spin effects, and whether such spin effects also play a significant role remains unclear. Furthermore, the contribution magnitude of spin effects in different catalyst systems, as well as how they influence the spin‐forbidden OER/ORR processes, remain to be further investigated and clarified. Thus, the mechanism underlying the role of spin factors in OER/ORR has gradually emerged as a subsequent research focus, which is expected to serve as a design principle and consideration for next‐generation high‐efficiency catalysts. Elucidating how spin influences oxygen‐involved reactions is anticipated to be the key to breakthroughs in catalytic performance, thereby providing a new dimension for the design of outstanding performance electrocatalysts.

**Figure 1:**
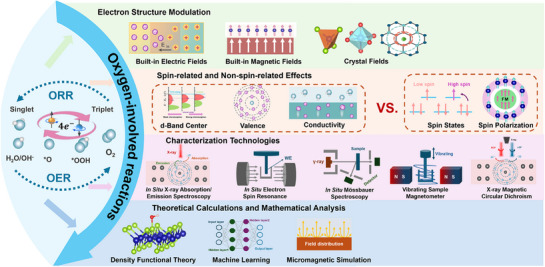
Outline of decoupling spin‐related and non‐spin‐related effects from electronic structure modulation for oxygen electrocatalysis.

In this review, we summarize the spin‐related and non‐spin‐related effects from electronic structure modulation during OER/ORR process and clarify their individual mechanism and coupling effects on catalytic performance. Importantly, we highlight the decoupling of spin‐related and non‐spin‐related effects for oxygen‐involved reactions via *in/ex situ* characterization, theoretical calculations and mathematical analysis (**Figure** **1**). By systematically analyzing the mechanisms and synergistic effects of spin and non‐spin factors from electronic structure modulation for oxygen‐involved reactions (OER/ORR), this work aims to provide new perspectives, approaches, and methods for the design and optimization of spin‐controlled electrocatalysis.

## 2 Strategies of Electronic Structure Modulation

In the exploration of efficient non‐precious metal catalysts for oxygen‐involved reactions, researchers typically employ the strategies of electronic structure modulation, including built‐in electric fields, built‐in magnetic fields, crystal fields, and ligand fields. Common experimental approaches to achieve these modifications include doping, strain engineering, defect/vacancy introduction, heterostructure construction, surface reconstruction, coordination modification, and p‐n heterojunctions.

### 2.1 Built‐in Electric Fields

**Figure 2:**
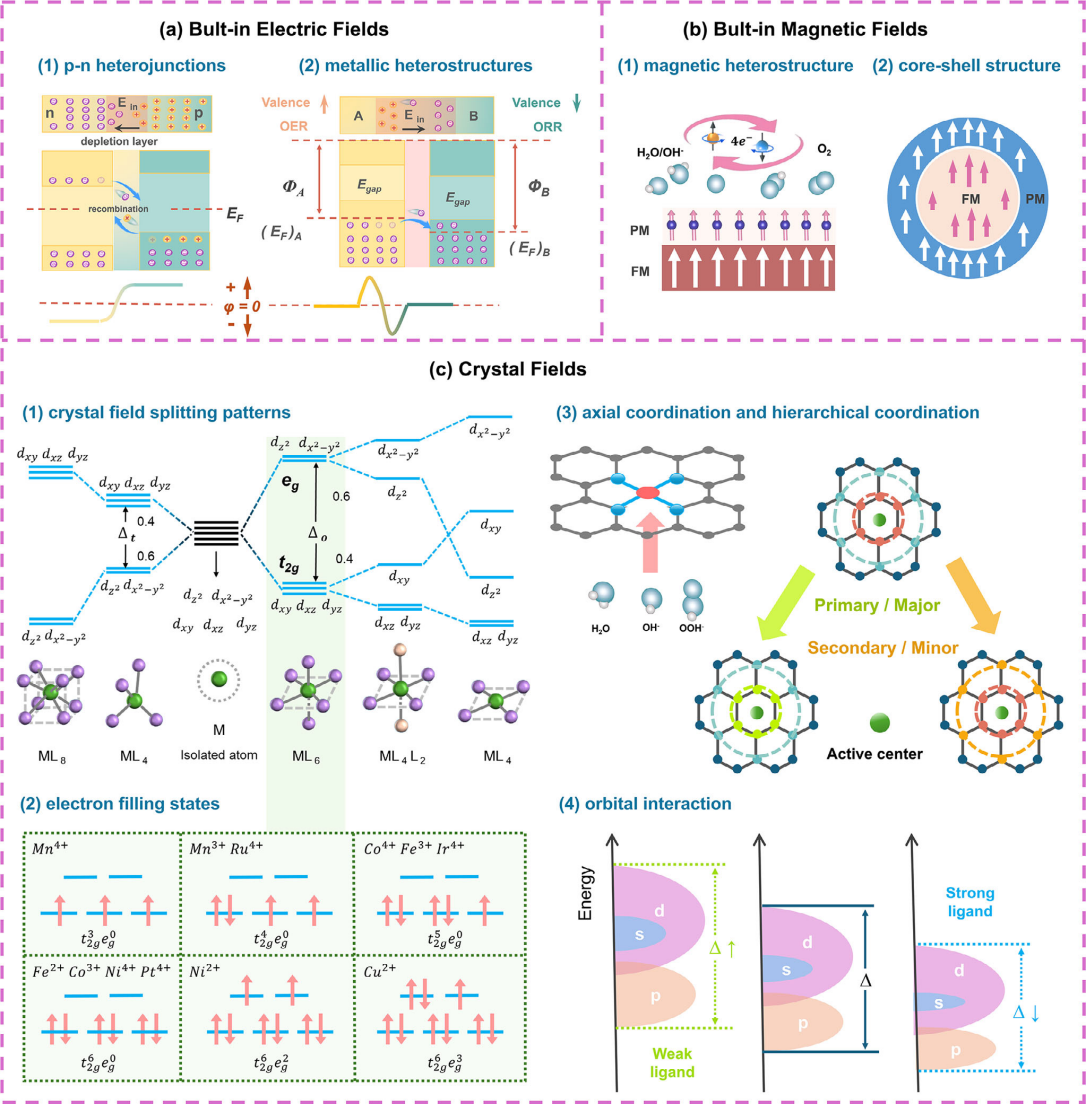
The four strategies of electronic structure modulation are: a) built‐in electric fields formed by p‐n heterojunctions or metallic heterostructures; b) built‐in magnetic fields created via magnetic heterostructure or core‐shell structure; c) crystal fields regulation adjusted through splitting patterns, electron filling states, axial coordination and hierarchical coordination and orbital interaction.

The construction of built‐in electric fields typically relies on differences in carrier types (p‐type and n‐type) (**Figure** **2**(a1)) and work functions (Figure 2(a2)) between two materials. Structural rearrangement and interfacial charge transfer facilitate the formation of nanoscale electric fields, which modulate the surface electronic structure of the material.^[^ ^59^ ^]^ Experimentally, metallic heterostructures represent a prominent strategy for establishing built‐in electric fields, significantly enhancing catalytic performance by promoting charge transfer and electronic structure optimization. When metallic materials with differing work functions form a heterostructure through mutual contact, electrons from the interface with the lower work function will transfer to that with the higher one. This method can be employed to achieve precise control over the valence state of the target catalyst. Notably, recent studies have highlighted the potential of p‐n heterojunctions in generating built‐in electric fields to promote spin‐polarized electron accumulation and transport, offering insights into the selective electron transfer mechanisms in spin‐sensitive oxygen‐involved reactions.

When a heterojunction forms between an n‐type semiconductor (with electrons as carriers) and a p‐type semiconductor (with holes as carriers), a depletion region arises at the interface after the diffusion equilibrium of movable carriers. Upon injecting an applied electric field into the current, carriers on both sides migrate in the low‐energy direction, forming a high‐speed electron transfer channel. If the injected current consists of single spin‐oriented electrons, surface electron spins align in parallel, which is more favorable for spin‐selective oxygen‐involved reactions. For example, Zhang et al.^[^ ^60^ ^]^ constructed a NiFe‐LDH/Co_3_O_4_ p‐n heterojunction on a nickel foil substrate. By injecting spin‐isotropic electrons from the nickel substrate to Co_3_O_4_ via an applied magnetic field, these electrons were then transferred to Fe/Ni sites in the surface layer of NiFe‐LDH through Co─O bonding, resulting in the spin‐parallel arrangement of electrons. The parallel alignment of electrons in the *σ anti‐bonds of two neighboring *OH was synergistically promoted by Ni and Fe, accelerating the generation of *OO and speeding up OER (with the overpotential reduced by 25 mV at 50 mA cm^−2^). Additionally, the built‐in electric fields generated by the p‐n junction affects the electronic structure of the surface catalyst, distorting it and thereby modulating the d‐band center and spin states. For instance, Li et al.^[^ ^34^ ^]^ utilized the p‐type semiconductor property of a single‐atom catalyst (MeN_x_) and constructed a p‐n heterojunction with an n‐type semiconductor substrate (gallium sulfide with a low work function). This induced distortion of the FeN_4_ structure and transition of the Fe^2+^ spin state through the interfacial space‐charge region, leading to a more than two‐fold enhancement of ORR activity. Furthermore, by modulating the work function with different substrates, they achieved linear and continuous regulation of FeN_4_ activity.

### 2.2 Built‐in Magnetic Fields

When one of the layers in heterostructures is a magnetic material, it can generate a built‐in magnetic field to polarize another layer to acquire spin‐parallel electrons, including planar magnetic heterostructure (Figure 2(b1)) and core‐shell structure (Figure 2(b2)). Magnetic materials can be categorized into ferromagnetic (isotropic magnetic moments, FM), antiferromagnetic (reversed and equal magnetic moments, AFM), ferrimagnetic (reversed and unequal magnetic moments, FiM), and paramagnetic (no long‐range magnetic order, PM) based on magnetic moment arrangement. In recent years, FM materials have frequently been used as substrates for oxygen‐involved reactions to study the role of spin polarization/pinning effects in catalysts. Unlike non‐magnetic heterostructures that require an external magnetic field or spin current device to assist the substrate in generating spin electron/current, magnetic substrates possess intrinsic magnetic moments (spin‐imbalanced electrons) that polarize surface catalysts while acting as spin filters to generate spin current. This allows electrons reaching the surface to align in spin‐parallel arrangement, promoting the spin‐inconsistent four‐electron transfer reactions.^[^ ^61^ ^]^

Experimentally, constructing magnetic core‐shell structures is an effective model to study the mechanism of spin polarization in oxygen‐involved reactions. For example, Xu et al.^[^ ^36^ ^]^ designed an FM‐AFM core‐shell structure Fe_3_O_4_@Ni(OH)_2_ to enhance spin electron separation and significantly improve OER kinetics by selectively removing spin‐opposed electrons. In recent years, FM‐PM heterostructures have emerged as a research hotspot, as the interfacial FM exchange field can penetrate the reconfigured hydroxyl oxide layer, activating the function as a spin filter and accelerating spin‐selective electron transfer.^[^ ^62^ ^]^

### 2.3 Crystal Fields

The crystal field theory arises from the perturbation of d‐orbitals (*d_xy_*, *d_xz_*, *d_yz_*, $d_{z^{2}}$, $d_{x^{2}-y^{2}}$) due to symmetry breaking in transition‐metal compounds, lifting orbital degeneracy and changing electron filling states. Orbital arrangement is determined by compound symmetry (e.g., octahedral field, tetrahedral field, square planar field) (Figure 2(c1)), and electron filling follows the principles of energy minimization, Pauli exclusion, and Hund's rule (spin‐parallel filling of equivalent orbitals to maximize total spin) (Figure 2(c2)).

The analytical framework is divided into three key steps: (1) structural symmetry determination (tetrahedron, octahedron, *D_4h_* point group, square planar, etc.); (2) symmetry‐adapted interactions analysis of all valence orbitals (*s*, *p_x_*, *p_y_*, *p_z_*, *d_xy_*, *d_yz_*, *d_xz_*, $d_{z^{2}}$, $d_{x^{2}-y^{2}}$)—covalent bonding model; (3) d‐orbital symmetry evaluation for active sites with crystal field splitting characterization—electrostatic model. Taking octahedral Co^4+^ (CoSe_2_) and tetrahedral Co^2+^ (CoSe) as representative examples, covalent bonding analysis is as follows: The outermost orbital configuration of Co and Se are 3d^7^4s^2^ and 3d^10^4s^2^4p^4^. Upon hybridization between Co(4s^2^) and Se(4p^4^), four equivalent sp^3^ hybrid orbitals are formed and populated with six electrons. The electrostatic consideration is following: In octahedral coordination, stronger electron repulsion within the d orbitals drives two electrons to transition into the sp^3^ orbitals, resulting in a tetravalent state (Co^4+^). In contrast, in tetrahedral coordination, electron repulsion in the d orbitals is less pronounced, precluding electron transition to the sp^3^ orbitals and thus retaining a divalent state (Co^2+^). Subsequently, the electronic ground state configuration is determined by considering the d orbital splitting patterns under different symmetries. Figure 2(c2) illustrates the electronic ground state configurations for various ions under the octahedral splitting pattern. Further consideration must be given to the relationship between splitting energy and electron pairing energy to determine other possible configurations.

To modulate the crystal fields, many experimental approaches can be employed, such as stress‐strain engineering, doping, and defect/vacancy introduction. Stress‐strain engineering regulates orbital splitting energy (*∆*) by altering bond length, thereby affecting d‐orbital arrangement and electron filling (e.g., Zou et al.^[^ ^63^ ^]^ found that IrO_6_ octahedral piezoelectric strain in Sr_2_IrO_4_ reduces the d‐band center, with temperature further continuously regulating the d‐band center to enhance OER activity). Doping affects both the crystal field splitting energy and valence state to affect d‐orbital alignment by introducing lattice distortion. For example, Kowalski et al.^[^ ^37^ ^]^ doped NiOOH with low‐concentration and low‐spin‐state Fe^3+^, shifting the d‐band center upward and reducing the OER overpotential from 0.72 to 0.42 V. Defect/vacancy introduction induces crystal field distortions by altering surface coordination environments (e.g., O/S/P/metal vacancies), affecting d‐orbital alignment. For instance, Liu et al.^[^ ^64^ ^]^ introduced Cr vacancies in Co_3_O_4_ with tensile strain, modulating the electronic structure to shift the d‐band center upward and reducing the OER overpotential to 327 mV at 10 mA cm^−2^. Furthermore, other methods include axial coordination and hierarchical coordination (Figure 2(c3)). For instance, Wang et al.^[^ ^65^ ^]^ induced d‐orbital splitting aberration through axial coordination in Fe−N_4_ at Fe sites, shifting Fe from a low spin state to a high spin state and facilitating *OH desorption to enhance ORR performance. Zou et al.^[^ ^67^ ^]^ used the secondary coordination effect of neighboring N═C─N groups to regulate the spin state for low spin (LS) to intermediate spin (IS) state of Fe active centers. Finally, ligand fields are a derivative of crystal fields, employed to describe orbital interactions within the ligand structures of amorphous organic molecules (Figure 2(c4)).

In summary, experimental methods such as heterostructures construction (p‐n heterojunctions, magnetic core‐shell structures, and planar magnetic heterostructures), doping, stress‐strain engineering, vacancy/defect introduction, coordination modification, and surface reconstruction enable the electronic structure modulation of catalysts. However, these modifications simultaneously alter multiple physicochemical descriptors, such as d‐band center position, valence state, conductivity, spin state, and spin polarization. Consequently, disentangling the individual contributions of spin‐related and non‐spin‐related factors remains a significant challenge.

## 3 Spin and Non‐Spin Effects from Electronic Structures

Despite significantly enhancing catalytic performance, these modulation strategies simultaneously alter multiple physical parameters, making it challenging to isolate individual contributions. Specifically, the mechanisms and synergistic interplay between non‐spin effects (e.g., d‐band center, valence state, conductivity) and spin‐related effects (spin state, polarization) remain unclear—constituting a key challenge in current research.

### 3.1 Non‐Spin‐Related Effects

In oxygen‐involved reactions, non‐spin effects overlook the spin dynamics inherent to the four‐electron transfer process. These effects principally include d‐band center position, valence state, electrical conductivity.

#### 3.1.1 d‐Band Center

**Figure 3:**
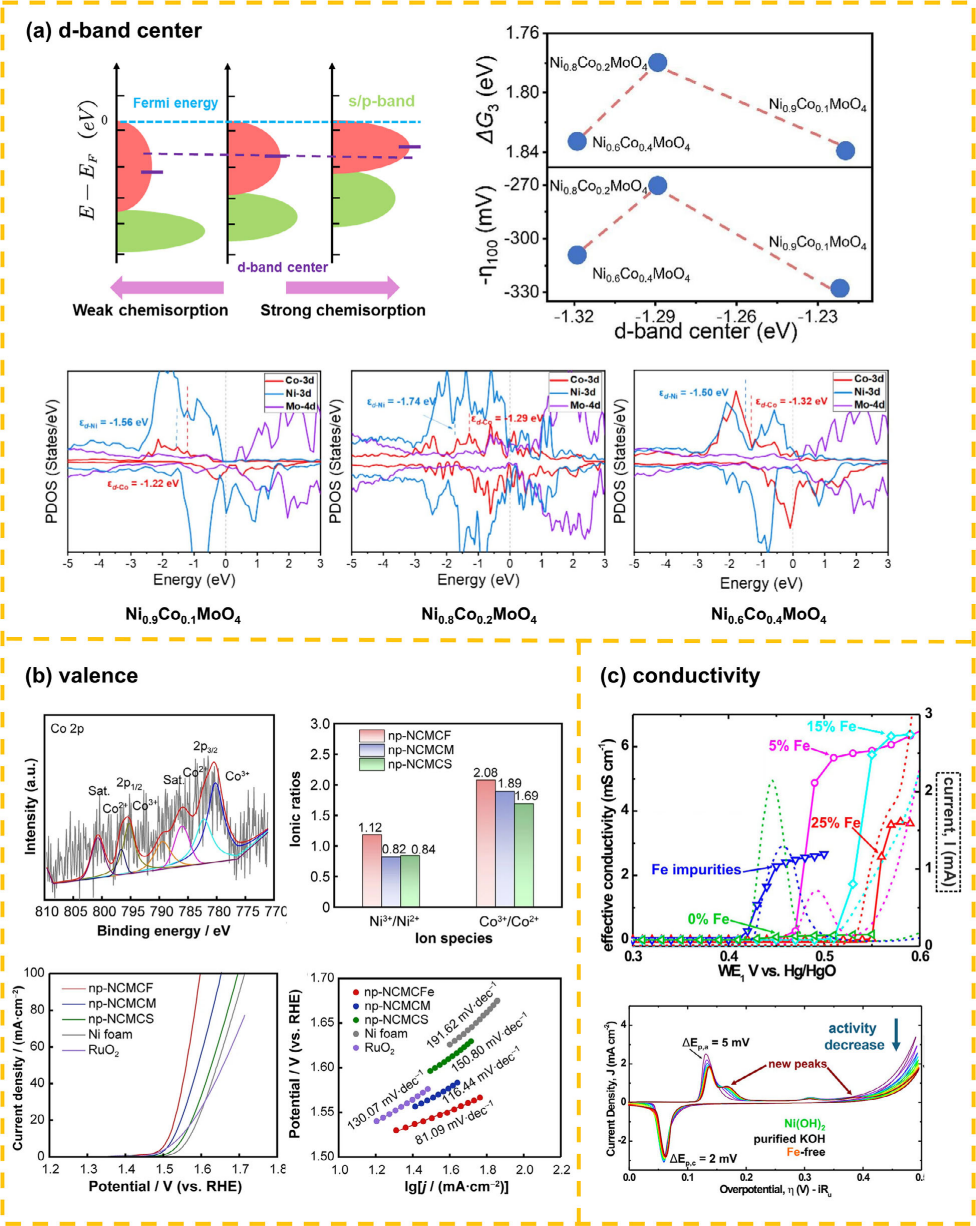
The non‐spin‐related effects from electronic structure modulation: a) d‐band center (Reproduced with permission.^[^ ^73^ ^]^ Copyright 2025, Elsevier), b) valence (Reproduced with permission.^[^ ^75^ ^]^ Copyright 2023, Springer Nature), c) conductivity (Reproduced with permission.^[^ ^78^ ^]^ Copyright 2014, American Chemical Society).

According to the Sabatier principle, optimal catalytic activity requires moderate adsorption strength of reaction intermediates—species that bind either too strongly or too weakly lead to inferior performance.^[^ ^5^ ^]^ The surface activity of transition metal compounds is primarily determined by d‐orbitals, and the d‐band center theory proposed by Nørskov et al.^[^ ^9^, ^70^, ^71^ ^]^ uses this as an intrinsic activity descriptor: lower (too weak adsorption) or higher (too strong adsorption) d‐band centers are detrimental, with moderate positions yielding the most active catalysts (in top left corner of **Figure** **3**a).

For example, Luo et al.^[^ ^66^ ^]^ used asymmetric geometry in a novel mesoporous Co−N_3_O single‐atom catalyst to localize more electrons around the Co center, shifting the d‐band center upward, stabilizing the *O intermediate and promoting ORR. Kang et al.^[^ ^72^ ^]^ utilized the curvature of carbon carriers to shorten Fe─N bond lengths, spatially redistributing charge around Fe and lowering the d‐band center to optimize adsorption of oxygen‐containing species (ORR half‐wave potential of 0.926 V). Jiang et al.^[^ ^73^ ^]^ modulated the d‐band center of nickel sites in NiMoO_4_ via cobalt doping to optimize the adsorption energy of OER intermediates and lower reaction energy barriers (NiCoMoO_4_·nH_2_O with an overpotential of 270 mV at 100 mA cm^−2^) (Figure 3a).

#### 3.1.2 Valence State

The valence state of active centers significantly impacts catalytic performance. Generally, high valence states favor OER, while low valence states favor ORR.^[^ ^74^ ^]^ Stabilizing high‐valence active sites by introducing non‐precious metals (e.g., Sc, Ti, V, Cr) to modify Fe/Co/Ni‐based catalysts can enhance OER performance (Figure 3b).^[^ ^75^ ^]^ For instance, Lu et al.^[^ ^76^ ^]^ prepared Cr‐Cu/CoO_x_ catalysts via an electrodeposition and annealing process, where Cr doping modulated Cu/Co high valence states, reducing the OER overpotential to 252 mV at 10 mA cm^−2^. Luo et al.^[^ ^77^ ^]^ investigated V/Cr/Mn/Cu‐doped CoOOH and found OER activity positively correlated with Co valence state (CoMnOOH > CoCrOOH > CoCuOOH > CoVOOH > CoOOH), with overpotentials of 256 mV, 313 mV, 329 mV, 322 mV, and 360 mV (at 10 mA cm^−2^), respectively.

#### 3.1.3 Electrical Conductivity

Early studies proposed electrical conductivity as a key factor, but subsequent research demonstrates that it is not a rate‐determining factor in oxygen electrocatalysis, as electron transport within catalysts is inherently rapid. Instead, kinetic limitations predominantly arise from proton transfer dynamics, orbital hybridization processes, or spin‐flip excitation barriers.^[^ ^8^, ^78^, ^79^, ^80^, ^81^ ^]^ For instance, Boettcher et al.^[^ ^78^ ^]^ observed a 30‐fold increase in conductivity for Ni_1‐x_Fe_x_(OH)_2_/Ni_1‐x_Fe_x_OOH films, yet activity changes could not be explained by conductivity alone, with electronic structure changes being central (Figure 3c). Additionally, adding conductive toner or using conductive substrates in tests largely mitigates conductivity effects; further, spin‐sensitive oxygen‐involved reactions require the parallel alignment of electrons, and traditional conductivity measurements cannot reflect spin currents (e.g., some oxides exhibit better conductivity than noble metals but still have insufficient catalytic performance^[^ ^8^, ^79^ ^]^). Xu et al.^[^ ^80^ ^]^ verified this in studies of magnetic field‐induced spin polarization on OER, showing conductivity has negligible impact on catalytic performance.

### 3.2 Spin‐Related Effects

Non‐spin effects, which do not account for the intrinsic spin magnetism of electrons, have limitations in describing spin‐dependent four‐electron transfer reactions. Modulating spin structures can break spin‐forbidden transition and linear constraints, fundamentally enhancing catalytic performance. Its core mechanisms include spin state regulation of adsorption/desorption dynamics and spin polarization modulation of electron‐selective transfer kinetics.

#### 3.2.1 Spin States

**Figure 4:**
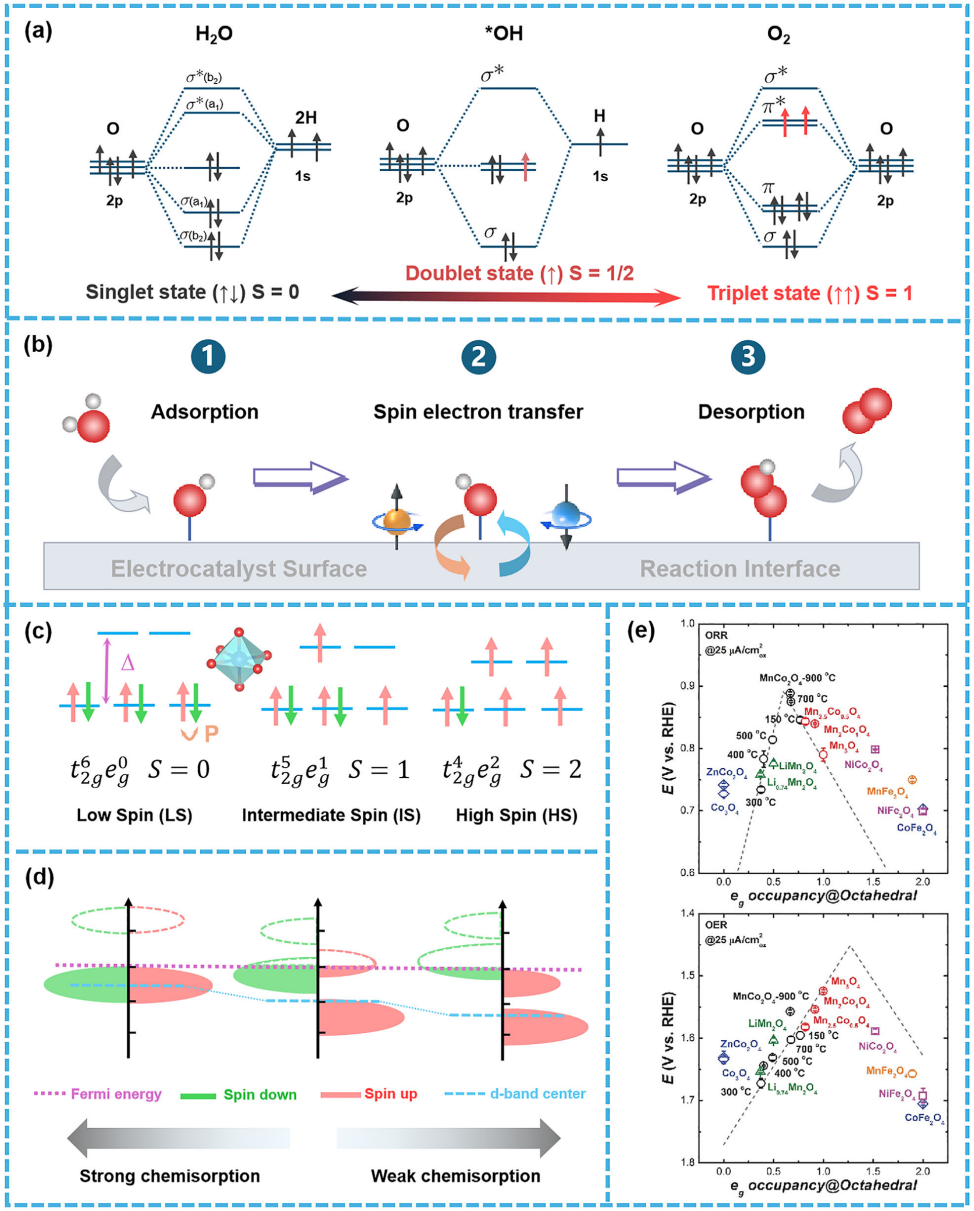
The variations of spin states from reactions and electrocatalysts. a) The change of spin states from singlet H_2_O/OH^−^ to triplet O_2_. b) The three‐step key reaction process on the electrocatalyst surface: adsorption, spin electron transfer and desorption. Schematic diagram of c) Spin state transitions and d) spin‐electron energy levels in octahedral catalytic centers. e) The relationship between OER/ORR catalytic activity and *e_g_* orbital occupation of octahedral center. Reproduced with permission.^[^ ^117^ ^]^ Copyright 2017, John Wiley and Sons.

For the OER and ORR, the spin state mismatch between reactants and products—characterized by the continuous transition between singlet water and triplet oxygen—renders these processes spin‐forbidden reactions (as illustrated in **Figure** **4**a). When occurring on catalyst surfaces, the reaction can be divided into three critical steps: adsorption of H_2_O/OH^−^, spin electron transfer between catalyst and intermediates, and desorption of O_2_ (as shown in Figure 4b). The overall reaction rate is determined by both the adsorption/desorption dynamics and the spin electron transfer kinetics.

Taking octahedral active centers as an example, symmetry breaking lifts the degeneracy of the five d‐orbitals. Under electrostatic repulsion from the surrounding eight coordinating atoms, the d‐orbitals split into two higher‐energy *e_g_* orbitals ($d_{z^{2}}$, $d_{x^{2}-y^{2}}$) and three lower‐energy *t_2g_* orbitals (*d_xy_*, *d_xz_*, *d_yz_*), with the energy difference defined as the orbital splitting energy (*∆*). For systems with six d‐electrons, three distinct filling configurations arise depending on the magnitude relationship between the electron pairing energy (*P*) and *∆*: (i) When *P*>*∆*, electrons fully populate the *t_2g_* orbitals ($t_{2g}^{6}e_{g}^{0}$), resulting in equal numbers of electrons with opposite spins and zero unpaired electrons, corresponding to the LS state. (ii) When *P* is slightly less than *∆*, one electron from the *t_2g_* orbitals jumps to populate the *e_g_* orbitals, leaving the *t_2g_* orbitals with 5 electrons ($t_{2g}^{5}e_{g}^{1}$). This configuration exhibits more spin‐up than spin‐down electrons (2 unpaired electrons), defined as the IS state. (iii) When *P*≪*∆*, both *e_g_* orbitals are fully populated, with the *t_2g_* orbitals containing 4 electrons ($t_{2g}^{4}e_{g}^{2}$). Here, spin‐up electrons far outnumber spin‐down electrons (4 unpaired electrons), designated as the high spin (HS) state. All electron configurations adhere to Hund's rule (Figure 4c).

The energy distributions of these spin states corresponding to different electron configurations are depicted in Figure 4d. The LS state, with the high‐energy empty orbital, exhibits strong adsorption of reaction intermediates, which is unfavorable for reaction progression. Conversely, the HS state, with the low‐energy empty orbital, shows weak intermediate adsorption, also impeding catalytic efficiency. By comparison, the IS state results in the moderate adsorption strength, thus facilitating reaction kinetics, consistent with the Sabatier principle of chemisorption.^[^ ^5^ ^]^ This observation is further supported by experimental studies, which have established a volcano‐type relationship between OER/ORR catalytic activity and *e_g_* orbital occupancy (Figure 4e).^[^ ^117^ ^]^

For instance, Zou et al.^[^ ^67^ ^]^ modulated the Fe spin state from LS to IS state through secondary coordination at Fe−N_4_ sites, finding that catalytic activity with *e_g_* = 1 was significantly enhanced (Fe_SAC_−NC mass activity was three times that of Pt/C); Hou et al.^[^ ^68^ ^]^ adjusted the Co^3+^ spin state from HS to IS state via Fe‐substituted LaCo_1‐x_Fe_x_O_3_ perovskites, resulting in a sixfold enhancement in OER activity. Unlike the d‐band center (which reflects average electron states), spin states modulation focuses on electron accumulation in a single spin orientation (magnetic moment), making it particularly suitable for spin‐sensitive reactions. The volcano plot relating catalytic activity to the number of *e_g_* electrons, proposed by Shao‐Horn's group (with IS state exhibiting optimal activity), has laid the groundwork for spin state regulation.^[^ ^1^ ^]^

Recent studies have deepened this understanding. Sun et al.^[^ ^29^ ^]^ induced a spin state transition of Fe^3+^ in NiFe‐LDHs via Cu doping, reducing the OER overpotential to 210 mV at 10 mA cm^−2^; Luo et al.^[^ ^77^ ^]^ observed that the number of Co^3+^ *e_g_* electrons within the range of 0.13 to 1.21 was inversely proportional to the OER overpotential (e.g., CoMnOOH with *e_g_* = 1.21 exhibited an overpotential of 256 mV, while CoOOH with *e_g_* = 0.13 showed 360 mV). Sun et al.^[^ ^81^ ^]^ synthesized axial hydroxyl ligand high‐spin Fe−N_4_ (S = 5/2), stabilizing the spin state via π interactions and significantly enhancing ORR activity. Emerging concepts and mechanisms related to spin states have also been explored. Ye et al.^[^ ^82^ ^]^ reported potential‐induced spin crossover from LS to HS state in MOF‐based catalysts, forming a shallow hole‐trap state and enhancing *OH/*O oxidation. Chen et al.^[^ ^83^ ^]^ observed spin crossover‐driven dimerization in diiron electrocatalyst [Fe_2_(µ‐O)(µ‐OH)(L_1_)_2_], which strengthened metal‐ligand covalent bonding and reduced the OER overpotential to 180 mV at 10 mA cm^−2^. Therefore, a suitable spin state is the key point for the optimal adsorption/desorption equilibrium.

From the above analysis, spin state modulation of adsorption/desorption dynamics is rationally validated by *e_g_* orbital occupancy. The catalyst design should aim to engineer active centers with appropriate spin state (macroscopically manifests magnetic moments) to achieve optimal the adsorption strength of intermediates. Notably, spin state modulation is not unique; its regulatory strategy is analogous to that of the d‐band center and is not exclusive to oxygen‐involved reactions, as its essence lies in orbital interactions.^[^ ^84^, ^85^, ^86^, ^87^ ^]^ Thus, spin states are macroscopic quantitative descriptors of orbital electron occupancy, whose essence is the result of orbital interactions. However, it is significant to emphasize that this correlation between catalytic activity and *e_g_* orbital occupancy, derived from preliminary analysis of catalyst magnetism, serves as a descriptor for subsequent investigations into spin electron transfer kinetics. Furthermore, spin state discussions have focused primarily on the catalyst itself and have not addressed the initially highlighted spin‐forbidden transition of the oxygen‐involved reactions. Therefore, in addition to satisfying appropriate spin state requirements, the most critical function of catalysts is to facilitate selective electron transfer, i.e., spin polarization, as analyzed below.

#### 3.2.2 Spin Polarization

Oxygen‐involved reactions entail a four‐electron transfer process, characterized by the transition from singlet water to triplet oxygen. The reaction pathways in acidic or neutral solutions are outlined in Equations ((1), (2), (3), (4)), while those in alkaline solutions are detailed in Equations ((5), (6), (7), (8)). Taking the alkaline OER via the adsorption evolution mechanism as an example, the reaction proceeds through four key steps: First, OH^−^ adsorbs onto the catalyst surface and transfers a spin‐down electron, forming the active intermediate *OH. Second, the *OH intermediate further transfers a spin‐up electron with proton desorption, generating the second active intermediate *O. Third, *O adsorbs an additional OH^−^ and transfers a spin‐down electron, yielding the third intermediate *OOH. Fourth, *OOH undergoes a final spin‐down electron transfer with proton desorption to produce O_2_.

**Figure 5:**
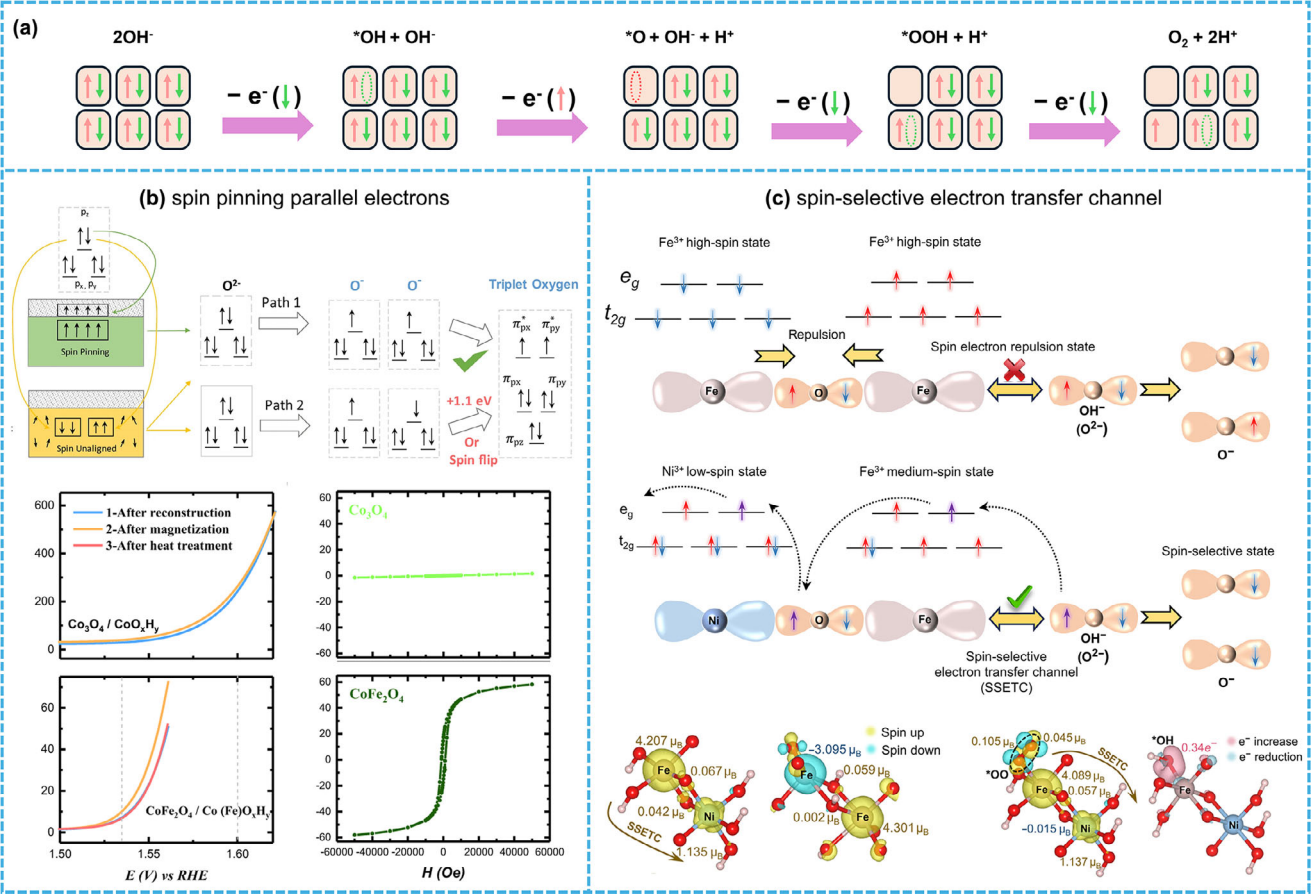
The kinetic process of spin polarization. a) A four‐electron transfer process of the alkaline OER via the adsorption evolution mechanism and the spin polarization effects: b) spin pinning parallel electrons (Open access^[^ ^4^ ^]^) and c) spin‐selective electron transfer channel (Reproduced with permission.^[^ ^69^ ^]^ Copyright 2025, The American Association for the Advancement of Science).

For such a spin‐forbidden reaction, two scenarios are required to reconcile spin state mismatches: either one of the four electrons undergoes a spin flip, or three electrons of identical spin and one of opposite spin are selectively transferred (as illustrated in **Figure** **5**a). Correspondingly, two strategies emerge to achieve spin electron transfer. The first involves enhancing the magnetism of the catalyst, leveraging spin pinning effects to induce spin flipping and parallel alignment of electrons, thereby facilitating O─O coupling and accelerating the reaction. The second strategy focuses on designing spin‐selective transfer channels to enable unimpeded transfer of spin electrons. Thus, spin polarization promotes spin‐sensitive reactions by optimizing electron transfer kinetics, which can be categorized into two mechanisms: spin pinning parallel electrons and spin‐selective electron transfer channel.

In acidic or neutral solutions:

(1) $$H_{2}O+*\to *\mathrm{OH}+H^{+}+e^{-}\left(↓\right)$$

(2) $$*\mathrm{OH}\to *O+H^{+}+e^{-}\left(↑\right)$$

(3) $$*O+H_{2}O\to *\mathrm{OOH}+H^{+}+e^{-}\left(↓\right)$$

(4) $$*\mathrm{OOH}\to *+O_{2}\left(g\right)+H^{+}+e^{-}\left(↓\right)$$

In alkaline solutions:

(5) $$OH^{-}+*\to *\mathrm{OH}+e^{-}\left(↓\right)$$

(6) $$*\mathrm{OH}+{\mathrm{OH}}^{-}\to *O+H_{2}O+e^{-}\left(↑\right)$$

(7) $$*O+OH^{-}\to *\mathrm{OOH}+e^{-}\left(↓\right)$$

(8) $$*\mathrm{OOH}+{\mathrm{OH}}^{-}\to *+O_{2}\left(g\right)+H_{2}O+e^{-}\left(↓\right)$$

##### Spin Pinning Parallel Electrons

Microscopic spin ordering (e.g., FM, AFM, FiM) in materials determines the manner of electrons parallel alignment.^[^ ^88^ ^]^ For instance, Ling et al.^[^ ^89^ ^]^ observed that FM ordering enhances Mn─O hybridization, accelerating electron transfer between surface Mn and adsorbed oxygen through spin‐up channels and thus improving ORR performance. Gracia et al.^[^ ^90^, ^91^ ^]^ proposed quantum excitation interactions (QEXI) and quantum spin exchange interactions (QSEI) to elucidate the microscopic mechanism of magnetic ordering for catalysts. Sun et al.^[^ ^29^ ^]^ utilized Cu^2+^ doping with Jahn‐Teller distortion to induce spin polarization from FiM to FM in NiFe‐LDHs. Xu et al.^[^ ^36^, ^79^, ^92^ ^]^ investigated Fe_3_O_4_@Ni(OH)_2_ core‐shell structures and found that an applied magnetic field can align the orientation of multi‐magnetic domains (single domains do not require an external magnetic field), thereby optimizing electron transport (single‐domain FM materials exhibit the most intrinsic energetic efficiency). They also explored the spin pinning effect at FM‐PM interfaces, which aligns PM hydroxyl oxide spins, lowers the O−O coupling barrier, and enhances the OER activity of Co_x_Fe_3‐x_O_4_.^[^ ^4^ ^]^ The specific mechanism is as follows:

During the alkaline OER catalysis process, Co_3_O_4_ undergoes surface reconstruction to form a hydroxide oxide layer (Co_3_O_4_/CoO_x_H_y_). Owing to the weak magnetism of bulk Co_3_O_4_, its spin pinning effect on the surface reconstructed layer is negligible, failing to effectively align spin electrons in parallel. As a result, both spin‐up and spin‐down electrons can be transferred. In this scenario, the intermediate active O^2−^ may form either spin‐down O^−^ or spin‐up O^−^ after electron transfer, which hinders the generation of triplet O_2_ from a statistical thermodynamics’ perspective. When a portion of Co is substituted with Fe, the magnetism of the Co_3‐x_Fe_x_O_4_ catalyst is significantly enhanced. Notably, for CoFe_2_O_4_ with an optimal doping level of x = 2, the catalyst transitions from paramagnetism to ferromagnetism, accompanied by a marked increase in magnetization. Under the spin pinning effect of the FM CoFe_2_O_4_ matrix, the intermediate active O^2−^ exclusively forms active O^−^ with a single spin state after electron transfer, thereby promoting the coupling of triplet O─O and accelerating the reaction (Figure 5b).^[^ ^4^ ^]^

This analysis exhibits a clear positive correlation with the enhancement of catalytic activity, and the spin flip energy of electrons is determined to be 1.1 eV. This may also account for the superior OER performance of iron‐based catalysts. Consequently, in catalyst design, emphasis should be placed on reasonably enhancing the magnetism of the matrix.

##### Spin‐selective Electron Transfer Channel

Maintaining magnetic order requires strong correlation and localization of d‐orbital electrons; thus, electron‐selective migration and orbital interactions are critical, necessitating the design of transfer channels with electron spin selectivity.^[^ ^11^, ^93^ ^]^ For instance, Xu et al.^[^ ^26^ ^]^ designed spinel LiCoVO_4_ to enhance OH^−^ spin electron removal and promote triplet O_2_ generation via spin‐selective transfer orbitals. Zou et al.^[^ ^62^ ^]^ exploited the symmetry breaking of the Fe─O─Ni structure in ZnFe_2‐x_Ni_x_O_4_ to construct FM ordered transfer orbitals, enhancing OER activity by fivefold. Ma et al.^[^ ^28^ ^]^ discovered that the spontaneous formation of spin‐polarized channels during the transition from SrCoO_2.5_ (triangular AFM) to SrCoO_3_ (cubic FM) significantly improved OER/ORR performance. Recently Dai et al.^[^ ^69^ ^]^ leveraged the synergy between IS Fe^3+^ sites and electron selective transfer in asymmetric Fe‐doped NiPS_3_ to boost seawater OER activity. The mechanism is explained as follows: Theoretical calculations reveal that when two adjacent active sites are occupied by Fe, they exhibit a high‐spin AFM state, with magnetic moments of −3.095 *µ_B_* and 4.301 *µ_B_* for the two Fe centers, respectively. Their orbitals are fully occupied with opposing electron spins; upon OH^−^ adsorption, electronic repulsion and non‐transferable orbits collectively hinder spin electron transfer. By contrast, substituting one Fe site with Ni induces a FM state: the Fe center exhibits a magnetic moment of 4.207 *µ_B_*, while the Ni center shows 1.135 *µ_B_*, with partially occupied orbitals that strongly facilitate the transfer of electrons with identical spins. This work characteristically incorporates and analyzes both spin pinning parallel electrons from neighboring sites and spin‐selective transfer channel aspects (Figure 5c).^[^ ^69^ ^]^ Notably, chiral materials and chiral engineering offer alternative approaches to constructing spin‐selective channels. For instance, recently topologically homochiral PdGa exhibits a current of 156 mA at 0.85 V, 100 times higher than non‐chiral PdGa.^[^ ^94^ ^]^ Research on utilizing chirality for spin‐selective channel design dates back to 2015, later extended to 2D chiral materials.^[^ ^17^, ^95^, ^96^ ^]^ Recent efforts have focused on spin manipulation via coupling of spin, charge, orbital, and lattice degrees of freedom.^[^ ^97^ ^]^

Taken together, for oxygen‐involved reactions, spin‐related effects cannot be neglected due to the spin non‐conservation inherent in their four‐electron transfer process. Such considerations must encompass two critical aspects: spin state modulation of adsorption/desorption dynamics and spin polarization modulation of spin‐selective electron transfer kinetics. Herein, spin state serves as a quantitative descriptor introduced to analyze the impacts induced by spin effects, with a primary focus on spin polarization to investigate the underlying mechanism. However, the dynamic processes and mechanistic insights by which spin polarization promotes oxygen‐involved reactions remain elusive, and the activity contribution arising from spin polarization requires further investigation.

### 3.3 Multi‐Factor Coupling Analysis

Multi‐factor synergistic modulation studies have revealed correlations between non‐spin‐related and spin‐related effects. For example, Luo et al.^[^ ^67^ ^]^ investigated V/Cr/Mn/Cu‐doped CoOOH and found that OER overpotential is inversely proportional to both the number of *e_g_* electrons and valence state. Plenty of literature indicates that the influence weight of spin and non‐spin effects from electronic structure modulation requires further decoupling to clarify dominant factors.

Based on extensive literature data synthesis, the same distribution trend and no significant regularity was observed in catalytic performance induced by single‐variable modulation (Figures S1 and S2, Supporting Information). Further analysis was conducted under three scenarios: spin effects alone, non‐spin effects alone, and their combined consideration.

**Figure 6:**
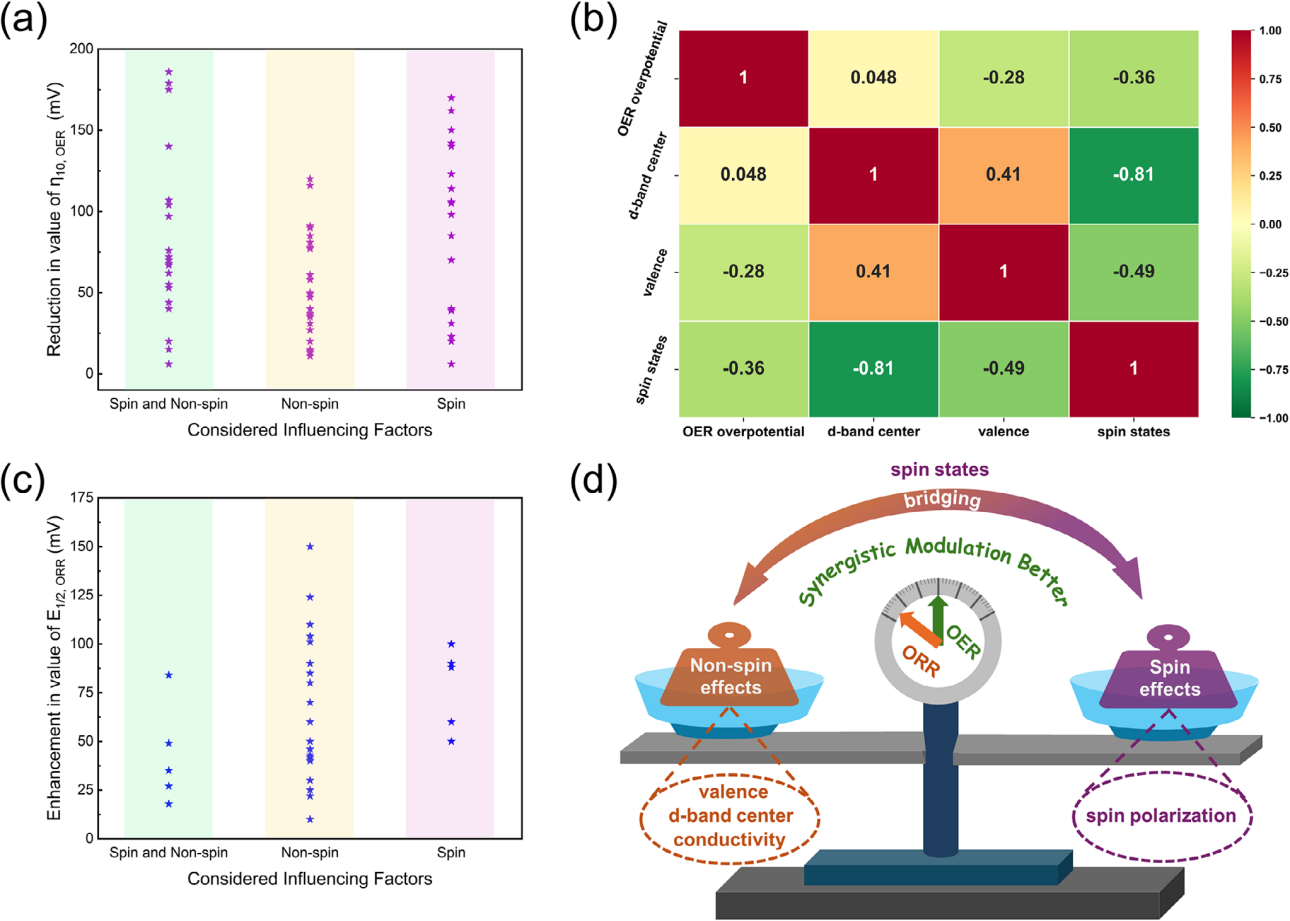
The influence of spin and non‐spin effects from electronic structure modulation for OER and ORR. a) The reduction in value of OER overpotential considered influencing factors. b) The covariance correlation matrix heatmap of the different variables for OER performance using machine learning. c) The enhancement in value of ORR half‐wave potential considered influencing factors. d) The schematic illustration of the influence and synergistic modulation for spin and non‐spin factors on OER/ORR catalytic performance.

For OER, it can be observed from **Figure** **6**a that when considering only spin factors, the reduce in OER overpotential exhibits a slightly greater advantage compared to considering only non‐spin factors. Furthermore, simultaneous consideration of both spin and non‐spin factors results in a marginal additional improvement relative to their individual effects. A preliminary conclusion can be drawn that spin and non‐spin factors hold comparable significance for OER, and their synergistic modulation is more conducive to the improvement of catalytic performance. Thus, optimal catalytic performance requires initial optimization of valence state and d‐band center, followed by modulation of spin state and polarization, enabling synergistic tuning to achieve optimal performance.

Notably, spin state regulation complements d‐band center effects in optimizing adsorption dynamics, as evidenced by their strong negative correlation (−0.81, Figure 6b). While d‐band center undergoes continuous tuning, spin state modulation involves discrete integer changes (as shifts in LS, IS and HS states), representing more drastic electronic perturbations. The relationship between the d‐band center and the energy level distribution of different spin states is consistent with that analyzed in Figure 4d. Furthermore, optimal spin state governs spin polarization kinetics by promoting the spin‐selective electron transfer, which may account for the higher activity enhancement by isolated spin effects (Figure 6a). Therefore, the synergistic effect for spin and non‐spin factors is important for the catalytic performance, which require the decoupling of spin and non‐spin effect for better designing of high‐performance catalyst in the future.

In contrast to the OER trend, the non‐spin factor plays a more pivotal role than its spin counterpart in enhancing the ORR half‐wave potential. This leads to a preliminary conclusion for ORR: non‐spin effects are the dominant contributor, yielding a more significant advantage over both isolated spin effects and their synergistic modulation. Notably, statistical analysis indicates that approximately two‐thirds of the studied systems are single‐atom catalysts (SACs) with transition metals anchored on nitrogen‐doped graphene (TM‐N‐C). The weak spin effects observed in these systems stem from insufficient spin polarization. This insufficiency impedes the full utilization of spin‐selective electron transfer, consequently resulting in negligible enhancement in catalytic activity from spin effects.

Therefore, the relative contribution of spin and non‐spin factors is catalyst‐dependent. While spin‐related and non‐spin‐related effects are comparably important in OER, with their synergy leading to superior performance, non‐spin effects dominate in ORR. This dominance arises from the weak spin polarization prevalent in SAC systems, which results in insignificant spin‐non‐spin synergy, as schematically illustrated in Figure 6d. Guided by these findings, we propose distinct design strategies for OER and ORR catalysts. For OER, the catalyst matrix should be engineered to exhibit strong spin polarization, thereby fully leveraging the kinetics of spin‐selective electron transfer. This, when synergized with the dynamics of adsorption/desorption inherent to non‐spin effects, can optimally enhance catalytic performance. Future efforts should thus focus on developing high‐spin‐polarization catalysts while maintaining favorable adsorption/desorption dynamics, for instance, by strategically enhancing catalyst magnetism. For ORR, the limited spin polarization in SAC systems restricts the contribution from spin‐selective electron transfer, rendering spin effects marginal. Therefore, future research should explore two promising strategies: (i) inducing ferromagnetic coupling via coordination engineering (e.g., in Fe‐Co dual sites) to promote spin polarization, and (ii) modifying catalysts with clusters that possess intrinsic strong spin polarization, such as those involving 5d heavy metals. In conclusion, both spin and non‐spin effects are pivotal, yet their synergistic mechanisms require deeper understanding. Elucidating their relative significance and underlying interplay is essential for providing precise guidance in the rational design of high‐performance catalysts.

## 4 Analysis Methods

Recent studies reveal that spin effects and non‐spin factors are equally critical for modulating catalytic activity. However, the synergistic mechanism between these effects—and the quantitative contribution of spin‐degree‐of‐freedom modulation to oxygen‐involved reactions remains unresolved. Herein, characterization techniques (e.g., in situ absorption/emission spectroscopy, in situ electron paramagnetic resonance, in situ Mössbauer spectroscopy, in situ vibrating sample magnetometry, X‐ray magnetic circular dichroism) and theoretical calculations/mathematical analysis methods (e.g., density functional theory, machine learning, finite element simulations) prove crucial.

### 4.1 Characterization Technologies

**Figure 7:**
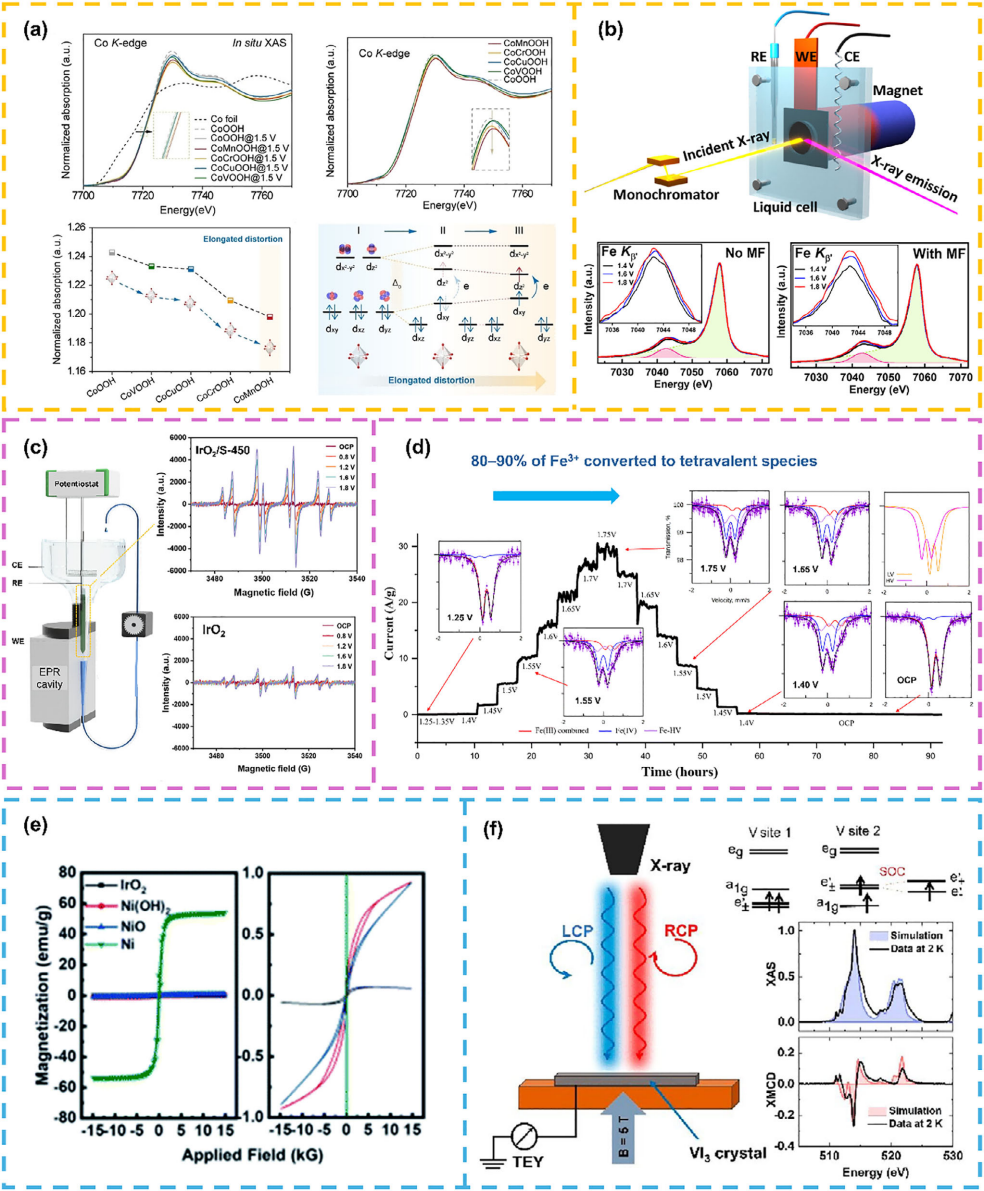
The *in/ex situ* characterization technologies for decoupling spin and non‐spin effects from electronic structure modulation: a) In situ X‐ray absorption spectroscopy decouples the impact of spin states and valence in V/Cr/Mn/Cu doping CoOOH on OER performance. Reproduced with permission.^[^ ^77^ ^]^ Copyright 2024, John Wiley and Sons. b) In situ X‐ray emission spectroscopy investigates the correlation between atomic/electronic structure, spin state evolution, and OER catalytic activity in CoFe_2_O_4_ catalysts under an applied magnetic field. Open access.^[^ ^98^ ^]^ c) The test set‐up of in situ electron paramagnetic resonance (left) (Open access^[^ ^99^ ^]^), and the spectroscopy of intermediates for IrO_2_/S‐450 and IrO_2_ during water splitting (right) (Reproduced with permission.^[^ ^30^ ^]^ Copyright 2025, John Wiley and Sons). d) The in situ ^57^Fe Mössbauer spectroscopy to study the influence of Ni/Fe valence in NiFe (8:1 molar ratio) aerogel catalysts. Reproduced with permission.^[^ ^103^ ^]^ Copyright 2025, Royal Society of Chemistry. e) The vibrating sample magnetometry to probe the OER behaviors of nickel‐based catalysts with distinct magnetic properties Ni(OH)_2_, NiO, and Ni under applied magnetic fields. Reproduced with permission.^[^ ^104^ ^]^ Copyright 2022, Royal Society of Chemistry. f) The X‐ray magnetic circular dichroism to quantify the orbital component of total magnetic moment in VI_3_. Open access.^[^ ^106^ ^]^

The advancement of *in/ex situ* characterization technologies provides critical support for analyzing the microscopic mechanisms of spin and non‐spin effects from electronic structure regulation. By tracking the dynamic evolution of catalyst structure and electronic states in time/space during reactions, these techniques effectively differentiate the contributions of the two effects and reveal their synergistic patterns (**Figure** **7**).

#### 4.1.1 In situ X‐Ray Absorption Spectroscopy (XAS)

In situ X‐ray absorption spectroscopy (XAS) monitors the dynamic evolution of valence state during electrocatalytic reactions by measuring X‐ray absorption coefficient variations with energy. It resolves catalyst chemical valence and electronic structure using X‐ray absorption near‐edge structure (XANES) and analyzes neighboring atomic coordination environments (e.g., bond lengths, coordination numbers) via extended X‐ray absorption fine structure (EXAFS). This makes it the optimal method for inferring the spin state of metal cations in oxygen‐involved reactions, serving as a core tool for studying structure changes. For instance, Luo et al.^[^ ^98^ ^]^ systematically investigated the modulation of CoOOH spin states and valence by V/Cr/Mn/Cu doping and its impact on OER performance using in situ XAS, providing experimental evidence for decoupling spin and non‐spin effects (Figure 7a). For in situ XAS, spatial resolution is limited, making it difficult to distinguish differences in spin properties between surface active sites (e.g., steps, defects) of catalysts and bulk structures, requiring additional characterization to supplement.

#### 4.1.2 In situ X‐ray Emission Spectroscopy (XES)

In situ X‐ray emission spectroscopy (XES) detects characteristic X‐rays emitted from samples to reflect local electronic structures (e.g., d‐orbital filling states), complementing XAS. It further analyzes the electronic state changes of active sites and intermediate adsorption behavior in OER/ORR. Chen et al.^[^ ^99^ ^]^ utilized in situ XES to deeply investigate the correlation between atomic/electronic structure, spin state evolution, and OER catalytic activity in CoFe_2_O_4_ catalysts under an applied magnetic field, revealing the key role of spin polarization in reaction kinetics (Figure 7b). But for in situ XES, signal intensity is significantly weaker than that of XAS, resulting in insufficient sensitivity for low‐concentration samples, requiring optimization of catalyst elemental loading and signal acquisition parameters.

#### 4.1.3 In situ Electron Paramagnetic Resonance (EPR)

In situ electron paramagnetic resonance (EPR) (or electron spin resonance, ESR) is also an important means to characterize the magnetic and electronic properties of materials with unpaired electrons. It provides key parameters such as the number of unpaired electrons, metal cation orbital occupancy, and local magnetic moment, offering direct evidence for analyzing spin states and polarization. Additionally, in situ EPR captures radical intermediates (e.g., •OH, •O_2_^−^) generated during reactions by detecting unpaired electron signals, revealing multi‐electron transfer pathways in OER/ORR and the mechanism of reactive oxygen species generation. The spin states of reactive oxygen radicals (e.g., •O_2_^−^, •OOH) are tightly linked to catalyst spin polarization (e.g., magnetic catalysts stabilize •OOH via spin exchange interactions). We can use EPR to characterize catalyst magnetic properties and in situ EPR to monitor •OOH spin state changes, establishing correlations between spin effects and catalytic performance.

M. Roessler et al.^[^ ^99^ ^]^ pioneered the development of operando film‐electrochemical electron paramagnetic resonance (FE‐EPR) spectroscopy, enabling real‐time tracking of radical intermediates in surface‐immobilized electrocatalysts (e.g., nitroxide‐catalyzed alcohol oxidation) under ambient temperature and aqueous flow conditions (test set‐up in left of Figure 7c). This breakthrough facilitates sensitive and quantitative detection of paramagnetic active‐site species while resolving reaction kinetics with high precision. In September 2024, Zhu et al.^[^ ^100^ ^]^ employed in situ EPR to monitor reactive species during piezo‐catalytic ORR over oxygen‐vacancy‐rich BiOBr. The detection of superoxide radical (•O_2_^−^) signals revealed a H_2_O_2_ formation pathway analogous to photocatalysis, providing direct evidence supporting the energy band theory for the piezo‐catalytic mechanism. In December 2024, Wang et al.^[^ ^101^ ^]^ utilized in situ EPR to investigate the electrochemical etching of Fe‐Mn oxides, observing increased oxygen vacancy concentration (characteristic g‐factor = 2.004 peak) under reducing potentials and partial vacancy filling under oxidizing conditions. This dynamic vacancy evolution elucidates the dissolution mechanism of surface Mn‐enriched phases and its impact on catalytic stability.

Most recently, Yang et al.^[^ ^30^ ^]^ reported in situ EPR studies showing negligible radical signals at open‐circuit potential, while increasing applied potential induced strong 5,5‐dimethyl‐1‐pyrrolidine N‐oxide (DMPO) signals attributed to DMPO‐O_2_ adducts, confirming in situ generation of •O_2_^−^ during OER on IrO_2_/S‐450. In contrast, minimal •O_2_^−^ intensity in pristine IrO_2_ indicated negligible lattice oxygen oxidation, consistent with the adsorption evolution mechanism (in right of Figure 7c). Complementarily, Hao et al.^[^ ^102^ ^]^ observed a distinct signal at 1.665 V (vs RHE) in quasi‐in situ EPR spectra of β‐Co(OH)_2_ at 90 K, providing the first direct evidence for the formation of Co^4+^ species. For in situ EPR, limited by cavity size, commercial electrolytic cells are lacking, and custom micro in situ reaction cells need to be fabricated to meet testing requirements.

#### 4.1.4 In situ Mössbauer Spectroscopy (MS)

In situ Mössbauer spectroscopy (MS), based on the recoilless γ‐ray resonance absorption (Mössbauer effect) of atomic nuclei, enables precise characterization through measurements of chemical shifts (*δ*), quadrupole splitting (*∆_EQ_*), and magnetic hyperfine splitting (*∆_HFS_*). These parameters directly determine nuclear oxidation state, spin states, coordination symmetry, and magnetic ordering (e.g., FM, AFM, FiM). Its exceptional sensitivity to the local atomic environment of specific nuclei (e.g., ^57^Fe, ^119^Sn) renders it uniquely suited for probing dynamic evolutions of metal sites in magnetic materials.

In oxygen‐involved reactions, in situ MS plays a critical role in elucidating the electronic structure and magnetic properties of Fe‐based catalysts. For example, during OER on Fe‐based systems (e.g., FeOOH, Fe─N─C, LaFeO_3_), Fe sites undergo dynamic valence (Fe^2+^, Fe^3+^ and Fe^4+^) and spin state (LS, IS and HS) transitions with reaction potential variations. By real‐time monitoring of *δ* (oxidation state/electron cloud density) and *∆_EQ_* (ligand field symmetry), in situ MS uncovers correlations between spin state transformations and catalytic activity. For instance, P. Hermann et al.^[^ ^103^ ^]^ applied in situ ^57^Fe MS to study NiFe (8:1 molar ratio) aerogel catalysts, revealing that 80%–90% of Fe^3+^ converted to tetravalent species, persisting even when potential dropped below the OER onset. This work validated the pivotal role of high valent Ni and Fe in promoting OER (Figure 7d).

#### 4.1.5 In situ Vibrating Sample Magnetometer (VSM)

Vibrating sample magnetometry (VSM), a cornerstone technique for quantitative magnetic characterization of materials, operates through the interplay of mechanical sample oscillation and electromagnetic induction. By measuring magnetization responses, it yields precise magnetic moment data and deciphers spin ordering features (e.g., spin state, orbital occupancy). For instance, Xu et al.^[^ ^104^ ^]^ employed VSM to probe the OER behaviors of nickel‐based catalysts with distinct magnetic properties Ni(OH)_2_, NiO, and Ni under applied magnetic fields, elucidating the preeminent role of the magnetoresistance effect in governing apparent OER activity (Figure 7e).

Modern VSM systems, incorporating high‐sensitivity transducers and precise temperature regulation, enable operando measurements under reactive environments. This capability facilitates tracking dynamic evolution of metal center spin and oxidation states, thereby disclosing intrinsic correlations between catalytic activity and electronic structure. In oxygen‐involved reactions, in situ VSM provides unparalleled insights into the electron and atom structure dynamics of active sites. For transition metal oxide catalysts (e.g., Co_3_O_4_, MnO_2_), fluctuations in d‐electron occupancy directly govern the oxidation states of metal centers (e.g., Co^2+^/Co^3+^, Mn^3+^/Mn^4+^) and their associated magnetic moments. During OER, real‐time magnetization shifts monitored by VSM correlate with the formation/consumption of oxygen intermediates (e.g., *OOH, *O) and lattice oxygen activation. Through quantitative operando magnetic profiling, VSM not only supplements conventional spectroscopic techniques (e.g., XAS, EPR) but also uncovers cryptic magnetism‐reactivity correlations—accelerating the rational design of efficient, stable oxygen‐involved catalysts.

#### 4.1.6 X‐Ray Magnetic Circular Dichroism (XMCD)

X‐ray magnetic circular dichroism (XMCD) leverages the absorption disparity of circularly polarized X‐rays in magnetic materials (*∆µ* = *µ_L_* – *µ_R_*, where *µ_L_* and µ_R_ denote absorption coefficients of left/right circularly polarized light) and employs sum rules to quantitatively determine spin (*µ_S_*) and orbital (*µ_L_*) magnetic moments. As the sole technique capable of directly measuring spin polarization degree with exceptional sensitivity to spin‐orbit coupling (SOC) effects, XMCD enables quantitative exploration for dynamic correlations between SOC and magnetic moment orientation of active centers during reactions, thereby uncovering intrinsic links between electronic structure and magnetic properties.

Presently, XMCD has emerged as a pivotal tool for investigating magnetic 2D van der Waals materials, where magnetic properties are strongly modulated by local atomic geometry and environment.^[^ ^105^ ^]^ For instance, Piamonteze et al.^[^ ^106^ ^]^ utilized XMCD to quantify the orbital component of the total magnetic moment in VI_3_, identifying two distinct V sites with differentiated orbital occupancies and magnetic moment magnitudes, which directly corroborated the anomalous orbital magnetic moments of V^3+^ ions (Figure 7f).

When exploring the decoupling relationship between spin‐related and non‐spin factors from electronic structure modulation in oxygen‐involved reactions, as well as the mechanistic role of spin effects, conventional approaches and existing characterization techniques primarily focus on macroscopic analysis of bulk magnetic properties. However, catalytic reactions inherently occur at localized surface‐active sites, rendering these methods inadequate for unraveling microscale spin‐driven reaction mechanisms. Moreover, during spin manipulation, whether regulatory effects originate from the spin state change of active center—rather than perturbations from vacancies, defects, grain boundaries or crystal size—remains unvalidated.

In this scenario, the unique strengths of XMCD as a characterization tool are particularly pronounced: its capability to precisely probe element‐specific orbital and spin magnetic moments directly verifies the dominant role of active‐site spin changes in driving catalytic processes. For magnetic catalysts (e.g., NiFe_2_O_4_, Fe_3_O_4_, single‐atom Fe/Co/Ni/Cu─N─C), it monitors reaction‐induced spin magnetic moment variations at metal active sites, correlating these with intermediate (*O, *OOH) adsorption energies and overpotentials—a critical area demanding further development. For instance, Li et al.^[^ ^32^ ^]^ employed XMCD spectroscopy to probe Mn‐RuO_2_ nanoflakes, where characteristic signals at the Mn‐L_2_,_3_ and Ru‐L_2_,_3_ edges confirmed FM coupling between Mn^2+/3+^ and Ru^4+/5+^ ions, following the Goodenough‐Kanamori rule. Through sum rules analysis and configuration interaction calculations, the spin magnetic moments of Mn (0.15 *µ_B_*/Mn) and Ru (0.05 *µ_B_*/Ru) were quantified, unveiling the mechanism by which Mn doping triggers the transition of RuO_2_ from antiferromagnetism to room‐temperature ferromagnetism. This finding provides key evidence for magnetic field‐enhanced acidic OER activity, with the overpotential of 143 mV at 10 mA cm^−2^. But for in situ XMCD, testing needs to be conducted in a vacuum environment, and currently only quasi‐in situ characterization can be achieved; transparent‐window in situ cells need to be developed for real‐time monitoring.

*In‐situ* XAS is particularly effective for tracking the evolution of non‐spin factors (e.g., valence state and coordination environment) during the OER process. Meanwhile, techniques including *in‐ situ* AES, VSM, EPR, and XMCD can probe dynamic spin‐related changes, such as spin states and magnetic moments. Simultaneously, in situ infrared and Raman spectroscopy, along with differential electrochemical mass spectrometry (DEMS), offer insights into the structure and evolution of reaction intermediates. These advanced in situ characterizations, when combined with real‐time electrochemical measurements (e.g., current density, Tafel slope, and electrochemical impedance spectroscopy), provide a multidimensional perspective for decoupling and understanding synergistic mechanisms, where non‐spin and spin factors collectively regulate catalytic performance. However, these advanced *in‐situ* measurements are often challenged by experimental interference from the electrolyte environment, applied current, bubble formation, and vacuum conditions, making accurate detection during OER particularly difficult. Thus, understanding the synergistic mechanism between non‐spin and spin effects remains a significant challenge.

### 4.2 Theoretical Calculations and Mathematical Analysis

Beyond *in/ex situ* characterization, theoretical calculations and simulations and mathematical analysis bridge experimental phenomena and microscopic mechanisms, deepening understanding of complex experimental observations through quantitative analysis of crystal and electronic structure effects on oxygen‐involved reactions.^[^ ^107^ ^]^

#### 4.2.1 Density Functional Theory (DFT)

Density functional theory (DFT) reveals the electronic structure of catalyst active sites (e.g., d‐orbital filling, adsorption energy) by solving electron density/wave function as a function of energy, enabling in‐depth discussions of catalyst conductivity, reactivity, and stability‐related performance descriptors.^[^ ^108^, ^109^, ^110^ ^]^ In heterogeneous electrocatalytic oxygen‐involved reactions, DFT primarily follows the theoretical framework proposed by Nørskov, guiding catalyst design at the microscopic level by calculating Gibbs free energies of reaction intermediate adsorption (e.g., *O, *OH, *OOH), localizing active sites and quantifying reaction overpotentials.^[^ ^9^, ^71^ ^]^

**Figure 8:**
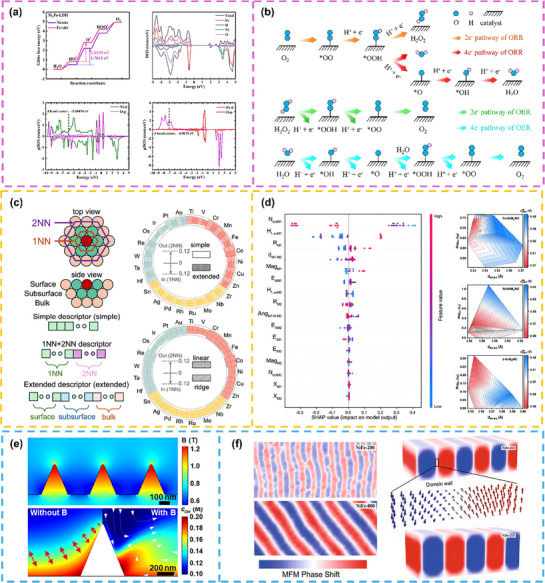
Theoretical calculations and mathematical analysis for decoupling spin and non‐spin effects from electronic structure modulation: Density functional theory calculation a) confirmed Ni in NiFe‐LDH species act as main active sites for OER (Reproduced with permission.^[^ ^110^ ^]^ Copyright 2025, Elsevier) and b) investigated the catalytic performance of a novel class for 2D single‐atom catalysts (Reproduced with permission.^[^ ^111^ ^]^ Copyright 2025, Elsevier). Machine learning c) accurately predicted the d‐band center values of disordered multi‐principal‐element alloys by capturing local coordination environments (Reproduced with permission.^[^ ^113^ ^]^ Copyright 2025, American Chemical Society) and d) explored magnetic moment as the second most important descriptor after structural factors in N‐bridged single‐atom catalysts (Open access.^[^ ^114^ ^]^). Micromagnetic simulations confirmed that the magnetic fields enhance the delivery of O_2_ reactants to the catalyst surface and expedite the removal of OH^−^ products, effectively addressing mass transport limitations in ORR by e) Finite element analysis (FEA) (Reproduced with permission.^[^ ^115^ ^]^ Copyright 2025, John Wiley and Sons), and showed that the reduction in magnetic domain walls promotes the spin‐sensitive OER via f) Finite Difference Method (FDM) on NiFe catalysts with varying thicknesses (Open access.^[^ ^116^ ^]^).

For instance, Li et al.^[^ ^110^ ^]^ used DFT calculations to confirm that Ni in NiFe‐LDH species act as main active sites for OER (**Figure** **8**a). Long et al.^[^ ^111^ ^]^ systematically investigated the catalytic performance of a novel class for 2D single‐atom catalysts via DFT, finding that among 30 transition metals embedded in C_4_N_3_ monolayer candidates, Cu−C_4_N_3_ exhibits optimal performance through a four‐electron mechanism, with ORR and OER overpotentials as low as 0.45 and 0.46 V, respectively (Figure 8b).

#### 4.2.2 Machine Learning (ML)

Machine learning (ML) leverages large datasets from DFT calculations and experiments, using models such as Random Forest Regression (RFR) and Extreme Gradient Boosting (XGBR) to screen key descriptors (adsorption energy, d‐band centers, geometric structure, etc.). It quickly evaluates the weights of different factors on catalytic performance, predicts catalytic overpotentials, and accelerates catalyst screening. For instance, Pan et al.^[^ ^112^ ^]^ employed DFT to calculate the OER/ORR overpotentials in transition metal single‐atom‐anchored graphene systems, and utilized ML to conduct analysis of intrinsic descriptors, including *ΔG_ads_* (Gibbs free energy change of adsorption), *θ*_e_ (the number of d electrons), ICOHP (integrated crystal orbital Hamiltonian population), *Δ_Charge_* (charge transfer), *φ* (work function), and *ε* (d‐band center), in which the first two most critical and convenient.

Additionally, Ouyang et al.^[^ ^113^ ^]^ proposed a universal and physically interpretable model, encompassing 10680 DFT‐optimized surface layers and over 1.2 million sets of d‐band center data. By capturing local coordination environments, this model accurately predicted the d‐band center values of disordered multi‐principal‐element alloys (Figure 8c). Furthermore, Jiang et al.^[^ ^114^ ^]^ constructed an ORR catalytic “hotspot map” using geometry and electronic structure of diatoms as descriptors—an exemplar of geometric‐electronic coupling design—magnetic moment as the second most important descriptor after structural factors (Figure 8d), experimentally verifying the excellent performance of N‐bridged Co‐Mn single‐atom catalysts. Thus, it is interestingly phenomenon that the catalytic performance is widely observed enhancement when the active center atom is Co, Rh, or Ir with d^7^ structure, which is remarkably consistent with the spin effects discussed previously.

Thus, ML can be employed to decouple and analyze the contributions of spin effects in oxygen‐involved reactions. Specifically, a dataset correlating spin‐related descriptors with catalytic activity can be constructed, enabling comparative investigations with non‐spin parameters to evaluate the significance of spin effects. For instance, three candidate spin‐related descriptors are proposed herein: the magnetic moment (*µ*_B_), which quantifies the atomic magnetic moment and can be measured via EPR/VSM or calculated by DFT; the magnetic coupling constant (*J*), which describes intrinsic magnetic exchange interactions as derived from magnetic transition temperatures in field‐cooled curves; and the magnetic moment angle (*θ*), which reflects the polarization degree and can be obtained through DFT calculations or micromagnetic simulation.

#### 4.2.3 Micromagnetic Simulation

When investigating the specific role of spin‐related factors in oxygen‐involved reactions, this can be achieved via micromagnetic simulation, with the methods encompassing finite element analysis (FEA) and finite difference method (FDM).

For instance, Fu et al.^[^ ^115^ ^]^ simulated the local magnetic flux density and OH^–^ concentration distribution near the surface of Pt@Ni cone electrodes employing FEA (Figure 8e). The results showed that due to the conical morphology, the magnetic flux density at the tip of the Pt@Ni cone was significantly higher than that of flat structures; this enhanced magnetic field could significantly promote O_2_ molecule diffusion to active sites through the magnetohydrodynamic effect. A further comparison of OH^–^ concentrations with and without a magnetic field revealed that OH^–^ removal efficiency was significantly improved under the magnetic field (especially in the tip region), confirming that the magnetic field not only accelerates the delivery of O_2_ reactants to the catalyst surface but also promotes the desorption of OH^–^ products during ORR.

Furthermore, Xu et al.^[^ ^116^ ^]^ performed micromagnetic simulations on NiFe catalysts with different thicknesses using FDM, and found that magnetically ordered regions exhibited a stripe‐like morphology; as the sample thickness increased, the central magnetic domain area gradually decreased. This observation indicates that the enhanced catalytic activity stems from the disappearance of domain walls during magnetization: specifically, magnetization drives the evolution of the domain structure from a multi‐domain to a single‐domain state, where the surface area previously occupied by domain walls gradually vanishes and reorganizes into a uniform single‐domain configuration. Such structural evolution promotes spin‐sensitive OER (Figure 8f).

Thus, micromagnetic simulation also serves as an important analytical tool for investigating oxygen‐involved reactions; moving forward, it can be further utilized to decouple the contributions of spin effects by simulating the magnetic field distribution and changes on catalyst surfaces. Future simulations into the influence of spin polarization on catalytic reactions should address three core aspects: saturation magnetization of core/bulk materials (intrinsic magnetism); magnetic interactions between core/bulk materials and surface catalytic layers; and magnetic moment orientation of surface catalytic layers (degree of spin pinning). As outlined above, spin‐related parameters derived from simulations can be combined with ML for systematic analysis to enable in‐depth exploration and investigation.

## 5 Decoupling Strategies and Ideas of the Two Effects

**Figure 9:**
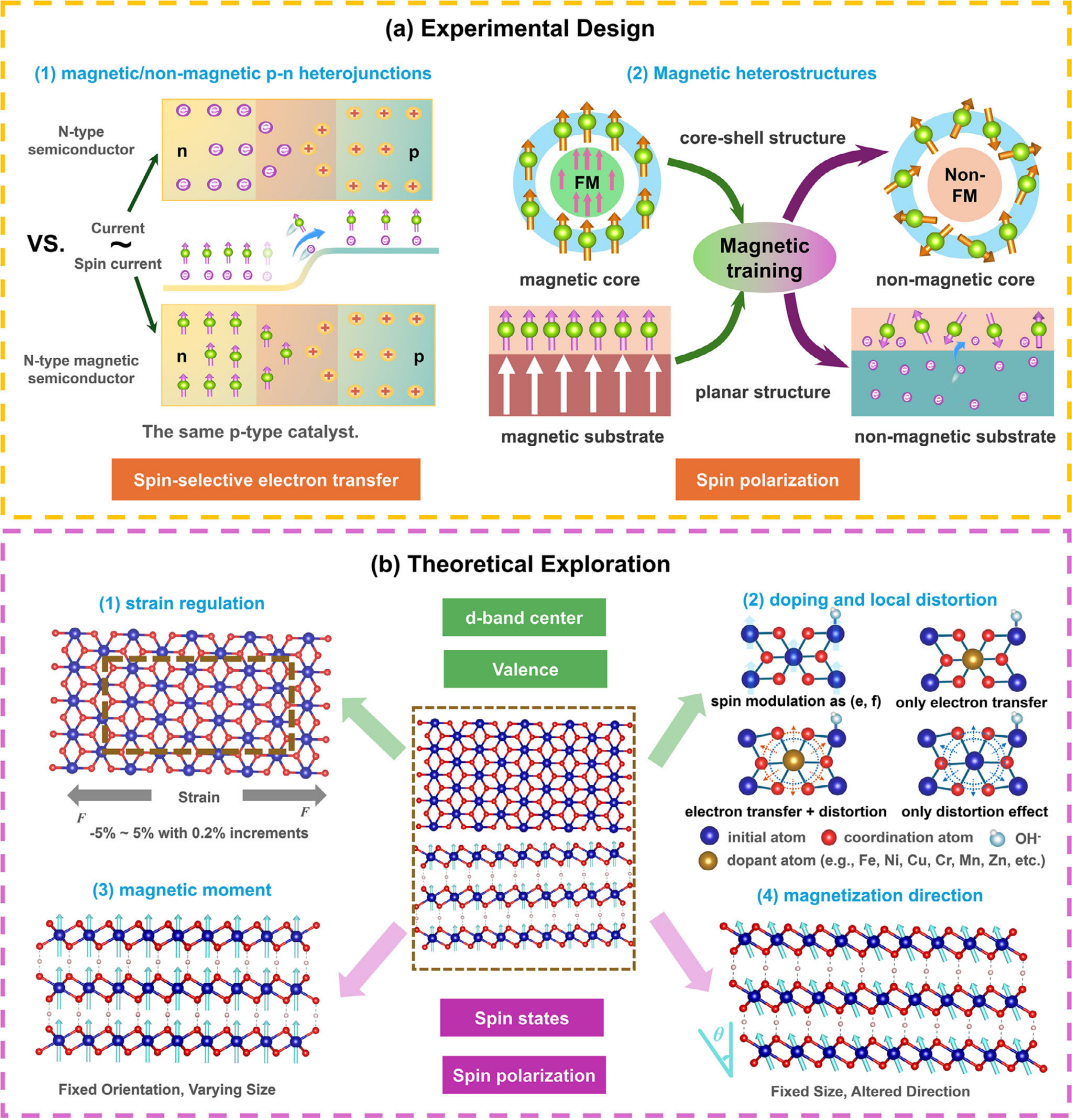
Decoupling strategies and ideas of the different effects from electronic structure modulation in OER/ORR. a) Experimental design for clarifying the significance of spin electron transfers kinetic and spin polarization by magnetic/non‐magnetic p‐n heterojunctions or by magnetic training for magnetic heterostructures. b) Theoretical exploration for distinguishing the influence of spin‐related effects (spin states and spin polarization) and non‐spin‐related effects (d‐band center and valence) by strain regulation, doping and local distortion calculation, magnetic moment regulation and magnetization direction modulation.

Based on prior analysis, this work proposes a decoupling strategy, which is from “experimental design” to “theoretical exploration”, focusing on the effects of surface spin state and internal spin polarization on catalytic performance (**Figure** **9**).

### 5.1 Experimental Design

Although experimental approaches such as heterostructures construction, stress‐strain engineering, doping, vacancy/defect introduction, coordination modification, and surface reconstruction often involve multi‐factor variations, mechanism‐oriented design can control single variables and reduce interference from other effects. Structural design and experimental treatment of catalysts enable the fixation of non‐spin factors, such as surface composition, lattice strain, valence, work functions, and conductivity, then focusing solely on the influence of spin factors on catalytic performance. Here fixing non‐spin factors via catalyst design and magnetic training treatment is as follows (Figure 9a):

1. Magnetic/non‐magnetic p‐n heterojunctions design. Maintaining the same surface p‐type catalyst, two distinct n‐type substrates—non‐magnetic and magnetic—are constructed, respectively, with spin current and conventional current applied to each to investigate the impact of electron transfer and spin‐selective transport on catalytic performance. When a p‐n heterojunction is formed, electrons and holes annihilate at the interface, resulting in the generation of a nanoscale depletion region. Upon application of an external voltage, electrons tunnel through the depletion region and migrate to the surface catalyst. When the n‐type material at one end serves as a magnetic substrate, electrons with identical spins accumulate; consequently, the electrons transported to the surface also exhibit uniform spin alignment, thereby promoting the reaction. Under such conditions, the interference caused by electron transfer driven by the built‐in electric field in conventional heterostructures can be avoided, thereby solely investigating the influence of spin factors on catalytic activity. (Figure 9(a1)).
2. Magnetic training and magnetization treatment. Systematically design different core‐shell combinations (e.g., FM‐core/PM‐shell, PM‐core/PM‐shell) to preliminarily explore the effect of internal magnetic field on catalysis. Magnetic training is employed by using alternating magnetic field or dynamic magnetization cycling to demagnetization and subsequent magnetization treatment is applied with an external magnetic field for constructed magnetic heterostructures. In this process, this method ensures that the chemical composition, crystal structure, morphology, and non‐spin electronic structure (e.g., *d*‐band center, *e_g_* occupancy) of the catalyst remain unchanged, with only the spin factors from substrate altered. Meanwhile, the electrochemical analysis should be focused on the kinetically controlled region of the polarization curves, where mass transfer effects of O_2_ are minimized. The comparative studies of catalytic activity before and after demagnetization can be employed to investigate the influence and mechanism from internal spin polarization for surface catalysts. Furthermore, the difference in activity enhancement between FM/PM and PM/PM core‐shell structures under the same magnetic field or demagnetization provides a quantitative measure of the additional spin polarization induced by the ferromagnetic core, excluding both the effect of mass transfer and built‐in electronic field (Figure 9(a2)).

These experiments should be combined with more *in/ex situ* characterization techniques (Figure 7) to track parameter dynamics during reactions: (1) In situ XAS to analyze active center valence, bond length, and coordination number evolution. (2) In situ XES to probe spin state (d‐orbital filling), coordination environment, and intermediate adsorption behavior. (3) In situ ESR to capture free radical intermediates (e.g., •OOH, •OH, •O_2_^−^) and analyze unpaired electron counts and their spin states. (4) In situ MS to track nuclear oxidation state, spin state, coordination symmetry, and magnetic ordering during reactions. (5) In situ VSM to monitor magnetic moment changes and analyze spin configurations. (6) XMCD to quantify dynamic correlations between active center spin‐orbit coupling and magnetic moment orientation, and active atom magnetic moment, revealing intrinsic electronic structure‐magnetic property relationships in electrocatalysts.

### 5.2 Theoretical Exploration

Spin‐polarized first‐principles calculations quantify how doping, strain, magnetic moment and spin polarizability modulate adsorption energy barriers, providing atomic‐level insights into decoupling spin/non‐spin catalytic tuning mechanisms. Multi‐variate DFT calculations can be designed to decoupling the individual contributions of various effects, such as electron transfer by doping, strain effect, and magnetic moment magnitude/orientation. Here weight quantification through theoretical exploration is as follows (Figure 9b):

1. Strain Regulation. Gradient‐tune lattice stretching in undoped pure‐phase materials (e.g., −5% to 5% with 0.2% increments), compute bond lengths and magnetic moments, deduce d‐orbital occupancy states, and specify spin configurations (strain modulates spin states by altering the balance between crystal field splitting energy and Coulomb repulsion energy). Quantify overpotentials to correlate strain effects with catalytic activity (Figure 9(b1)).
2. Doping and local distortion calculation. To isolate the effect of local lattice distortion in doped systems, it is crucial to maintain the local distortion rather than allowing a uniform change throughout the material. This is achieved through a four‐step computational approach that fully differentiates the impact of local distortion on catalytic performance. First, the spin modulation of initial atom is achieved in pure phase materials by manually adjusting the number of unpaired electrons (magnetic moment) or magnetization direction after structural optimization. Then, the incorporation of dopant atom (e.g., Fe, Ni, Cu, Cr, Mn, Zn) into pure phase structure without subsequent structural optimization is employed to demonstrate the effect of electron transfer from dopant atom. Subsequently, structural optimization of this doped structure is carried out, which introduces both electron transfer and lattice distortion effects. Finally, dopant atom is replaced by original host atom in the above optimized structure to retain the distortion effect while eliminating dopant‐electron transfer effect. Through the above four‐step comparative analysis, we can achieve complete differentiation of the various effects induced by local distortion from doping, including local distortion structure, d‐band center, spin state, electron transfer, etc. (Figure 9(b2)).
3. Magnetic moment regulation. By fixing atomic coordinates and magnetic moment orientation (either along the c‐axis or within the ab‐plane), systematically tune the atomic magnetic moment of the active center to investigate the effect of magnetic magnitude on catalytic performance (Figure 9(b3)).
4. Magnetization direction modulation. Isolate two distinct spin configurations (e.g., high‐spin, low‐spin) from systems with modulated strain/magnetic moments, fix magnetic moment strength while altering orientation (θ angle), compute overpotentials, and evaluate spin‐polarization kinetic effects (Figure 9(b4)).

Finally, by integrating machine learning techniques to analyze these computational data, we can quantify the weights of bond length, d‐band center, valence, spin state, and spin polarization on catalytic performance. The core of the spin manipulation strategy proposed herein lies in designing the optimal spin electronic structure of catalysts, promoting spin‐selective electron transfer during reactions by regulating the intrinsic magnetic properties of materials, and improving catalytic performance at the mechanistic level. It does not require structural modifications to existing large‐scale energy conversion devices compared with external magnetic field strategies that require additional integration of magnetic modules and are accompanied by significant energy consumption, it has higher application feasibility.

## 6 Conclusions and Perspectives

Electrochemical water splitting for hydrogen production, fuel cells, and rechargeable metal‐air batteries stand as core technologies in the new energy sector, dedicated to green hydrogen production, high‐efficiency energy conversion, and high‐capacity energy storage, respectively. However, their large‐scale deployment is hindered by a critical kinetic bottleneck of OER and ORR in electrocatalysis reactions. As the anodic reaction in water splitting and metal‐air battery charging, OER involves four‐electron transfer and complex intermediate conversion, leading to high overpotential and substantial energy consumption.^[^ ^16^, ^17^, ^18^, ^22^, ^24^ ^]^ Conversely, ORR, the cathodic reaction in fuel cells and metal‐air battery discharge, also suffers from slow kinetics, resulting in low energy conversion efficiency.^[^ ^19^, ^20^, ^21^, ^23^, ^25^ ^]^ Thus, breakthroughs in OER/ORR catalytic performance are pivotal to unlocking the commercial potential of these technologies.

As directed by above analysis, breakthroughs in OER/ORR performance not only directly address the core technical bottlenecks of water electrolysis, fuel cells, and metal‐air batteries but also accelerate the industrialization of new energy technologies by improving efficiency, reducing costs, and enhancing stability. Significantly, spin catalysis, as an emerging regulatory strategy, enables modulation of adsorption/desorption dynamics via spin state control and regulation of spin electron transfer kinetics through spin polarization—optimizing the spin matching of reaction intermediates and accelerating spin‐selective electron transfer. Thus, decoupling the roles of multiple key factors involved in OER/ORR processes (especially spin effects) and clarifying their respective microscopic mechanisms are imperative. This provides a novel paradigm for designing high‐efficiency OER/ORR catalysts, promising to break the limits of catalytic performance and ultimately drive the new energy system toward high economic viability, stability, and low environmental impact—serving as a core driver for global energy transition and the achievement of carbon neutrality.

By systematically synthesizing research progress on electronic structure modulation for oxygen‐involved reactions, we have summarized the roles of spin‐related and non‐spin‐related effects from electronic structure modulation and quantified the trends and contribution weights of multiple factors to catalytic performance using machine learning. Despite challenges in data collection comprehensiveness and accuracy arising from differing research focuses, we have preliminarily uncovered the overarching influence patterns of key factors.

Currently, research on spin catalysis is transitioning from “phenomenon discovery” to a full‐chain paradigm encompassing from “mechanism analysis” to “material design” and then to “device integration”. The core challenge lies in the unclear contribution ratios and mechanisms of spin and non‐spin effects, particularly the intrinsic enhancement mechanisms of catalytic performance by spin state and spin polarization regulation, which remain debated. Challenges also present opportunities, and future efforts should focus on the following directions:

1. Experimental design for decoupling spin and non‐spin effects. Targeted experimental models are required to clearly distinguish the mechanisms of spin and non‐spin effects from electronic structure modulation. For instance, simple and highly discriminative designs (e.g., core‐shell structures with spin‐regulated inner layers and non‐spin‐isolated outer layers, or gradient‐strain systems with continuous modulation of spin‐related parameters) are needed to minimize interference from non‐target factors. When complete experimental decoupling is challenging, theoretical calculations (e.g., DFT+U, time‐dependent DFT) and artificial intelligence (e.g., machine learning‐based feature screening) can be integrated to aid analysis, establishing a closed‐loop experiment‐theory‐data validation system.
2. Simple and controllable synthesis for magnetic catalysts. Magnetic materials are critical systems for studying spin effects in oxygen‐involved reactions. Traditional one‐step methods (e.g., solvothermal synthesis, electrodeposition) struggle to achieve precise synthesis, while mainstream techniques (e.g., chemical/physical vapor deposition, magnetron sputtering, high‐temperature sintering) face challenges of process complexity and harsh conditions. Future efforts should focus on developing simple, controllable synthesis techniques—such as microwave‐assisted synthesis, laser‐induced phase transition, and other rapid preparation methods—combined with microfluidic technology to enable composition gradient regulation, thereby controlling spin order, magnetic moment direction, and magnetic coupling strength at the atomic/nanoscale.
3. Highly time‐ and space‐resolved spin‐sensitive in situ characterization technology. In‐depth mechanism analysis relies on dynamic and in situ characterization tools. For oxygen‐involved reactions, urgent development is needed for spin‐sensitive, high time‐space‐resolved in situ techniques, with breakthroughs in: (i) time‐resolved in situ XAS and XES to track transient spin state evolution of active sites; (ii) spatially resolved in situ ESR and MS to visualize spin state changes; (iii) XMCD to quantitatively analyze dynamic correlations between spin‐orbit coupling and atomic magnetic moment magnitude/orientation during reactions, and in situ VSM to detect nanoscale. These techniques will form a “time‐space‐spin” 3D characterization network.
4. Fast and high‐precision spin polarization theory calculations and simulations. The complexity of magnetic structures, arising from the coupling of crystal symmetry and spin degrees of freedom, poses challenges to both accuracy and efficiency in spin polarization simulations using conventional quantum chemical methods and density functional theory. Future needs include: (i) symmetry‐based simplified models to reduce computational complexity; (ii) higher‐order generalized functionals incorporating spin‐orbit coupling (e.g., meta‐GGA, hybrid functionals) to improve accuracy in calculating magnetic moments and spin states; (iii) multi‐scale simulations combining machine learning force fields and molecular dynamics to efficiently predict spin‐sensitive reaction pathways.
5. Artificial intelligence‐driven multiscale collaborative research. Artificial intelligence (AI) and machine learning (ML) have become core tools to accelerate catalytic research. Future priorities include: (i) building standardized catalytic databases integrating experimental (e.g., in situ characterization data, activity test data) and computational (e.g., DFT, molecular dynamics, finite element simulations) data; (ii) developing multi‐scale ML models to correlate the relationships from “atomic structure” to “spin” and then to “catalytic activity” and achieve quantitative decoupling of spin/non‐spin effects; (iii) combining with generative AI to design “reverse synthesis” strategies for rapid screening of catalysts with optimal spin regulation capabilities, promoting closed‐loop from “data driven” to “experimental validation” and then to “theoretical modification” studies.
6. Catalyst design for multi‐field synergistic regulation. Distinguishing spin and non‐spin effects is not only a fundamental scientific issue but also a basis for multi‐factor synergistic regulation. Future efforts should optimize spin and non‐spin factors via synergistic modulation of electronic structure (e.g., electric, magnetic, crystal, and ligand fields) to achieve thermodynamic and kinetic dual driving of reaction pathways. The goal is to develop “spin and non‐spin synergistic” catalysts with high activity (lower overpotential), high stability (inhibiting spin depolarization), and high selectivity (modulating intermediate electron transfer pathways).
7. Technology translation and application expansion. Beyond basic research, accelerating the technical implementation of spin catalysis is critical: (i) overcoming stability bottlenecks of spin catalysts in acidic environments, developing low‐cost preparation processes (e.g., electrochemical deposition, gas‐phase migration) to lower industrial application thresholds; (ii) designing spin‐polarized electrolyze systems to synergistically regulate electron transfer efficiency via magnetic/electric fields, driving innovation in green hydrogen production and energy storage; (iii) expanding spin‐regulation strategies to other spin‐sensitive reactions (e.g., N_2_/NO_3_^−^ reduction, CO_2_ reduction) to provide generalized guidance for multidisciplinary energy conversion technologies.

In conclusion, in‐depth investigations of spin catalysis will drive comprehensive innovation from fundamental theory to applied technology in electrocatalysis. It is imperative to advance the understanding of spin effects from “qualitative recognition” to “quantitative design” through the deep integration of “experiment‐theory‐data”. Ultimately, this will enable breakthroughs in highly efficient, stable, and sustainable energy conversion technologies.

## Conflict of Interest

The authors declare no conflict of interest.

## Supporting information



Supporting Information
