# Supplementary material for: Decoupling of Spin and Non‐Spin Effects From Electronic Structure Modulation for Oxygen Electrocatalysis
Source: Adv Sci (Weinh). 2025 Nov 8;12(46):e14540. doi: 10.1002/advs.202514540 (PMC12697873; doi:10.1002/advs.202514540)
Supplement: Supplementary file 1 — Supporting Information [file ADVS-12-e14540-s001.docx]

Supporting Information

**Decoupling of Spin and Non-spin Effects from Electronic structure Modulation for Oxygen Electrocatalysis**

*Yingliang Zhao, Zhen Ji, Xiang Xiao, Zhi Fang*, and Yanglong Hou**

Y. Zhao, Z. Ji, X. Xiao, Prof. Z. Fang, Prof. Y. Hou

School of Materials, Shenzhen Campus of Sun Yat-Sen University, Guangdong 518107, China

E-mail: fangzh76@sysu.edu.cn; hou@sysu.edu.cn

Prof. Y. Hou

School of Materials Science and Engineering, Peking University, Beijing 100871, China

1. **Statistical Methods**

In this review, to dissect the contribution weights and impact magnitudes of spin and non-spin effects arising from the strategies of electronic structure modulation in oxygen-involved reactions (OER/ORR), we systematically survey and synthesize a vast body of literature, quantifying the variations in key parameters upon regulation via diverse strategies and methodologies. The statistical framework is elaborated as follows:

**1.1. OER Catalysis**

For OER, the strategies of electronic structure modulation are categorized into built-in electric fields, built-in magnetic fields, crystal fields, and ligand fields. Experimental methodologies encompass doping, heterostructure construction, vacancy/defect introduction, stress-strain engineering, surface reconstruction, size control, and coordination modification. We statistically analyzed the material system of electrocatalysts, OER overpotential, d-band center, valence, spin state, and consideration of spin polarization before and after modulation (**Table 1**). Specifically:

OER overpotential is defined as the value measured at 10 mA cm^−2^ in 1.0 M KOH.

The d-band center is derived from density functional theory (DFT) calculations, uniformly retained to three decimal places.

Valence is determined via X-ray photoelectron spectroscopy (XPS) or synchrotron radiation near-edge absorption spectroscopy (XNEAS). If authors provide the proportion of different valence components, the average valence of metal active centers is calculated and retained to three decimal places; in cases of only qualitative descriptions (*e.g.*, “minor changes” or “no significant variation”), valence is assumed unregulated and computed from the material’s molecular formula, retained as integers.

Spin state is acquired through three approaches: (i) magnetic moment of active centers from DFT calculations. (ii) Magnetic moment of magnetic atoms in chemical formulas from magnetic characterization. Both retained to three significant figures. (iii) Qualitative analysis of d-orbital configuration (low/medium/high spin), retained as integers.

Consideration of spin polarization is evaluated based on experimental design (*e.g.*, chiral materials for spin channels, applied external magnetic field in electrochemical tests) and mechanistic discussions (*e.g.*, spin electron transfer kinetics, selective orbital participation).

**Table 1. The datasets of different parameters for OER electrocatalysts**

| **Electronic structure Strategy** | **Experimental Approaches** | **Material System** | | **η_OER_ (mV)*** | | **d-band center (eV)** | | **Valence** | | **Spin states**  **(*μ_B_*)** | | **Spin Polarization** | **Refs** |
| --- | --- | --- | --- | --- | --- | --- | --- | --- | --- | --- | --- | --- | --- |
|  |  | **Before** | **After** | **Before** | **After** | **Before** | **After** | **Before** | **After** | **Before** | **After** |  |  |
| Built-in Electric Fields | heterostructure | MnSe | MnSe@MWCN | 260 | 240 | − | − | 2.000 | 2.500 | 0 | 0.950 | no | [1] |
|  |  | Ag/NiSe_2_/SCQDs/NF | Ag/NiSe_2_/MnSe/SCQDs | 241 | 235 | -1.290 | -1.040 | 3.400 | 3.700 | 1.150 | 1.200 | no | [2] |
| Built-in Magnetic Fields | doping | Ni-S-FeO_x_ | Ni_9.5_Co_0.5_-S-FeO_x_ | 230 | 215 | − | − | 0 | 2 | − | − | yes | [3] |
|  | size | NiFe-800 | NiFe-200 | 320 | 250 | − | − | − | − | − | − | yes | [4] |
|  | heterostructure | Co_3_O_4_ | Co_3_O_4_/NiFe-LDH | 387 | 217 | − | − | − | − | 5.400 | 3.900 | yes | [5] |
|  |  | CoFe_2_O_4_ | CoFe_2_O_4_@CoFeMo_3_O_8_ | 426 | 240 | − | − | 3 | 2 | − | − | yes | [6] |
|  |  | MnFe_2_O_4_ | Ni/MnFe_2_O_4_ | 440 | 261 | 0.950 | 0.870 | 3.000 | 3.200 | 0.120 | 0.240 | yes | [7] |
| Crystal Fields | size | Bulk LiCoO_2_ | 80 nm LiCoO_2_ | 370 | 330 | − | − | 3 | 3 | 1.000 | 1.200 | no | [8] |
|  | reconstruction | Ni-Al | Ni-Fe | 308 | 253 | 0.860 | 2.390 | 3 | 4 | − | − | yes | [9] |
|  |  | Co_x_Mn_1-x_O_y_ | Co_0.9_Mn_0.1_O | 420 | 352 | − | − | 2 | 3 | 3 | 0 | no | [10] |
|  | stress-strain | (FeCoNiCrMn)_3_O_4_ | annealing (FeCoNiCrMn)_3_O_4_ | 385 | 345 | − | − | − | − | 3.600 | 2.840 | no | [11] |
|  |  | NiFe LDH | D-NiFe LDH | 240 | 209 | − | − | 2.250 | 2.540 | − | − | no | [12] |
|  |  | Sr_2_IrO_4_ | Sr_2_IrO_4_ under heating | 312 | 235 | -2.21 | -2.57 | 4.000 | 4.010 | − | − | no | [13] |
|  | doping | CoOOH | Fe-CoOOH | 354 | 192 | − | − | 3 | 3 | 0 | 1 | no | [14] |
|  |  | Ir NCs | 5% CoIr NCs | 260 | 220 | − | − | − | − | − | − | yes | [15] |
|  |  | Fe_2_O_3_ | Co@Fe_2_O_3_ | 480 | 410 | 0 | 0.500 | 3 | 3 | 0 | 3.420 | yes | [16] |
|  |  | Fe_3_O_4_ | s-Fe_3_O_4_ | 290 | 270 | − | − | − | − | − | − | yes | [17] |
|  |  | Co-DABDT | Fe-DABDT | 411 | 306 | − | − | − | − | 2.080 | 3.140 | yes | [18] |
|  |  | CoFe-LDHs | NiCoFe-LDHs | 372 | 230 | − | − | − | − | − | − | yes | [19] |
|  |  | Fe−N_4_-Gra / Ti_2_NO | Co−N_4_-Gra / Ti_2_NO | 470 | 300 | − | − | − | − | − | − | yes | [20] |
|  |  | Co 3 O4 | ( Co ( 3-x ) Fex O4 ) | 341 | 318 | − | − | − | − | − | − | yes | [21] |
|  |  | CoOOH | Mo-CoOOH | 338 | 260 | − | − | 3.000 | 2.800 | − | − | no | [22] |
|  |  | Y_2_Ru_2_O_7-_*_δ_* | Y_2_Ru_1.6_Ir_0.4_O_7-_*_δ_* | 244 | 238 | − | − | 3.660 | 3.740 | 0 | 1 | yes | [23] |
|  |  | Fe−N−C | Mg/Fe−N−C | 347 | 224 | − | − | 2 | 2 | 2.960 | 0.950 | no | [24] |
|  |  | RuO_2_ NFs | Mn-RuO_2_ NFs | 267 | 200 | − | − | 4 | 5 | 0.045 | 0.050 | yes | [25] |
|  |  | NiO@NF | (Ru-Ni)O_x_@NF | 400 | 303 | − | − | 2.000 | 2.290 | 2 | 1 | yes | [26] |
|  |  | CoSe_2_ | Fe_0.4_Co_0.6_Se_2_ | 346 | 270 | -3.520 | -2.140 | 4.000 | 4.670 | 1 | 2 | no | [27] |
|  |  | Ni_3_S_2_ | Ni_3_S_2_-W-Vs | 352 | 246 | − | − | − | − | 3 | 2 | no | [28] |
|  |  | RuO_2_ | Mn_0.4_Ru_0.6_O_2_ | 235 | 196 | − | − | 4 | 4 | 0 | 1.250 | no | [29] |
|  |  | LaCoO_3_ | F_0.2_-LaCoO_3_ | 530 | 390 | -0.987 | -0.976 | 2.615 | 2.750 | 0 | 1 | no | [30] |
|  |  | NiPS3 | Fe/NiPS3 | 417 | 242 | − | − | 3.000 | 3.370 | 1.760 | 4.290 | no | [31] |
|  |  | Ni DHBQ/NF | FeNi DHBQ/NF | 292 | 207 | − | − | 3 | 3 | 0 | 3 | no | [32] |
|  |  | CoOOH | CoMnOOH | 360 | 256 | − | − | 3.000 | 3.500 | 0.130 | 1.210 | no | [33] |
|  |  | NiOOH | Fe doping NiOOH | 770 | 420 | -4.690 | -4.360 | − | − | 2 | 0 | no | [34] |
|  |  | NiCuMnCoSn | NiCuMnCoFe | 319 | 283 | − | − | 2.717 | 2.762 | − | − | no | [35] |
|  |  | NiCo_2_O_4_/NCNTs | MnCo_2_O_4_/NCNs | 400 | 363 | -1.250 | -1.040 | 2.515 | 2.507 | − | − | no | [36] |
|  |  | NiCo_2_O_4_/NCNTs | CoCo_2_O_4_/NCNTs | 400 | 350 | -1.250 | -1.150 | 2.515 | 2.490 | − | − | no | [36] |
|  |  | NiCo_2_O_4_/NCNTs | CuCo_2_O_4_/NCNTs | 400 | 353 | -1.250 | -1.230 | 2.515 | 2.474 | − | − | no | [36] |
|  |  | NiCo_2_O_4_/NCNTs | ZnCo_2_O_4_/NCNTs | 400 | 365 | -1.250 | -1.580 | 2.515 | 2.598 | − | − | no | [36] |
| Ligand Fields | heterostructure | NiFe-LDH | Fe_3_O_4_@C@NiFe-LDH | 249 | 196 | -1.318 | -1.821 | 3.0 | 3.2 | − | − | yes | [37] |
|  | defect/  vacancy | Ni - NDA | DD-Ni-NDA nanosheets | 410 | 260 | − | − | 2 | 2 | 1 | 0 | no | [38] |
|  |  | Co_3_O_4_ | E-Cr-Co_3_O_4_ | 388 | 327 | -3.210 | -2.930 | 2.620 | 2.610 | − | − | no | [39] |
|  |  | HEO | HEO-Zr_1.0_ | 270 | 257 | -5.205 | -4.717 | 4.000 | 4.500 | − | − | no | [40] |
|  |  | LMCO-1 | LMCO-2 | 435 | 420 | -3.090 | -2.670 | 2.540 | 2.570 | 1.170 | 1.010 | no | [41] |
|  | reconstruction | Co@CoO | Co@CoO-L | 340 | 300 | − | − | − | − | − | − | yes | [42] |
|  |  | Fe_5_Ni_4_S_8_ | Fe_5_Ni_4_S_8_-SDS | 352 | 245 | − | − | 2 | 3 | 4 | 5 | no | [43] |
|  |  | NiFe-MOFs | γ-NiFeOOH | 250 | 130 | -1.878 | -1.670 | 2 | 3 | − | − | no | [44] |
|  |  | MoS_2_/CNS | MoCoS/CNS | 546 | 263 | -1.653 | -1.357 | 4.500 | 4.900 | − | − | no | [45] |
|  |  | CuNi nanoalloy@N/C | Ni-CuO/CNO | 300 | 180 | − | − | 1.200 | 1.900 | − | − | no | [46] |
|  | coordination modification | CoFe_2_O_4_ | chiral molecule-modified | 470 | 430 | − | − | − | − | − | − | yes | [47] |
|  |  | RuO_2_ | chiral additives | 361 | 330 | − | − | − | − | − | − | yes | [48] |
|  |  | Fe−N−C | Mg-Fe-N-C | 347 | 224 | − | − | 2 | 2 | 2.960 | 0.950 | no | [49] |
|  |  | ZnCoVO_4_ | LiCoVO_4_ | 390 | 320 | − | − | 3 | 3 | 0.000 | 1.500 | no | [50] |
|  |  | Co-N-HPC | Co-NSP-HPC | 450 | 360 | -0.956 | -1.602 | 2.070 | 2.270 | − | − | no | [51] |
|  |  | NiCo LDH | NiCo LDH-TPA | 348 | 267 | -1.780 | -2.050 | 2.200 | 2.500 | − | − | no | [52] |
|  |  | Ni-MOFs | NiCo-MOFs | 287 | 243 | -2.000 | -0.880 | 2.000 | 2.300 | 3.190 | 2.960 | no | [53] |
|  |  | Ni-MOFs | NiFe-MOFs | 287 | 215 | -2.000 | -0.940 | 2.000 | 2.400 | 3.190 | 2.520 | no | [53] |
|  |  | Ni-MOFs | NiMn-MOFs | 287 | 325 | -2.000 | 0.260 | 2.000 | 2.000 | 3.190 | 2.040 | no | [53] |
|  |  | Ni-MOFs | NiZn-MOFs | 287 | 368 | -2.000 | -5.830 | 2.000 | 2.000 | 3.190 | 3.980 | no | [53] |
|  |  | Ni-MOFs | NiCu-MOFs | 287 | 281 | -2.000 | -3.090 | 2.000 | 2.200 | 3.190 | 3.450 | no | [53] |
|  |  | NiOOH | FeOOH/NiOOH | 210 | 195 | -1.650 | -1.770 | 2.500 | 2.650 | − | − | no | [54] |
|  |  | Cu-DBC | Cu/Co-DBC | 526 | 251 | -2.180 | -1.310 | 1.140 | 1.060 | − | − | no | [55] |
|  |  | Ru@FeMOF | Ru@Cr─FeMOF | 201 | 188 | -1.290 | -1.500 | 3.000 | 2.800 | − | − | no | [56] |
|  |  | RhSi | RhBiS | 335 | 221 | − | − | − | − | − | − | yes | [56] |
|  | doping | Fe-BHT | NiFe_1.4_-BHT | 412 | 350 | -0.170 | -0.059 | − | − | 1.940 | 2.300 | no | [57] |
|  |  | CoIr NCs | CoIr NC/M | 260 | 220 | − | − | 4.000 | 3.800 | − | − | yes | [58] |
|  |  | CuN_4_ - TOT | CoN_4_ - TOT | 420 | 280 | − | − | − | − | 1.410 | 1.280 | yes | [59] |
|  |  | NiFe LDHs | S-NiFe LDHs | 352 | 254 | − | − | − | − | − | − | yes | [60] |
|  |  | Cu/CoO_x_ | Cr−Cu/CoO_x_ | 289 | 252 | − | − | 2 | 3 | − | − | no | [61] |
|  |  | FeP@NBC | CoFe@NBC | 320 | 300 | -1.870 | -1.690 | 2.000 | 2.500 | − | − | no | [62] |
|  |  | NiFe | Ni(OH)_2_/FeOOH/Ag | 225 | 211 | − | − | 3.000 | 2.500 | − | − | no | [63] |
|  |  | Co-N-C | Cr-CoNC-1.00 | 450 | 410 | -1.500 | -1.320 | 2.000 | 2.500 | − | − | no | [64] |
|  |  | MoO_2_ | P-MoO_2_ | 365 | 280 | -2.150 | -1.630 | 4.000 | 3.500 | − | − | no | [65] |
|  |  | Co_3_O_4_ | In-Co_3_O_4_ | 433 | 406 | -2.446 | -2.391 | 2.500 | 2.510 | − | − | no | [66] |
|  |  | Co_3_O_4_ | Fe-Co_3_O_4_ | 433 | 384 | -2.446 | -2.543 | 2.500 | 2.530 | − | − | no | [66] |
|  |  | Co_3_O_4_ | Ni-Co_3_O_4_ | 433 | 422 | -2.446 | -2.786 | 2.500 | 2.520 | − | − | no | [66] |
|  |  | Co_3_O_4_ | Ga-Co_3_O_4_ | 433 | 437 | -2.446 | -2.795 | 2.500 | 2.470 | − | − | no | [66] |
|  |  | Co_3_O_4_ | Al-Co_3_O_4_ | 433 | 453 | -2.446 | -2.820 | 2.500 | 2.460 | − | − | no | [66] |
|  |  | RuO_2_ | P-RuO_2_ | 320 | 250 | -1.569 | -1.641 | 4 | 4 | 3 | 2 | yes | [67] |
|  |  | NiMoO_4_ | Ni_0.8_Co_0.2_MoO_4_·nH_2_O | 319 | 228 | -2.370 | -1.740 | 2.000 | 1.500 | − | − | no | [68] |
|  |  | NiS | VFe-NiS | 306 | 190 | -1.870 | -0.970 | 2.000 | 1.800 | − | − | no | [69] |
|  |  | Co_3_O_4_ | Mn-Co_3_O_4_ | 366 | 305 | -4.420 | -4.320 | 2.390 | 2.610 | − | − | no | [70] |
|  |  | Co_3_O_4_@CC | P‐Mo‐Co_3_O_4_@CC | 334 | 276 | -1.370 | -1.180 | 2.800 | 2.500 | − | − | no | [71] |
|  |  | Ni_3_S_2_ | SeFe-Ni_3_S_2_ | 480 | 330 | -1.550 | -1.640 | 2.000 | 1.800 | − | − | no | [72] |

* OER overpotential of catalysts at 10 mA cm^−2^ in 1.0 M KOH

**1.2. ORR Catalysis**

Like OER, the strategies of electronic structure modulation for ORR include built-in electric/magnetic fields, crystal fields, and ligand fields. However, experimental methodologies emphasize cluster loading, coordination modification, and stress-strain engineering, supplemented by doping, heterostructure construction, vacancy/defect introduction, surface reconstruction, and size control. Key parameters analyzed include catalyst composition, ORR half-wave potential, d-band center, valence, spin states, and consideration of spin polarization (**Table 2**). Notably:

ORR half-wave potential is the value measured in 0.1 M KOH.

The d-band center, valence, spin state, and consideration of spin polarization follow the same criteria as OER. For valence state determination, ORR studies predominantly focus on transition metal single-atom catalysts anchored on N-doped graphene, where qualitative descriptions (*e.g.*, “between 0–2 valence” or “slightly increased”) are common; in such cases, average values are adopted without considering valence modulation.

This systematic statistical framework aims to disentangle the intertwined spin and non-spin effects from electronic structure-modulated OER/ORR, providing quantitative insights for rational design of spin-engineered electrocatalysts.

**Table 2. The datasets of different parameters for ORR electrocatalysts**

| **Electronic structure Strategy** | **Experimental Approaches** | **Material System** | | **η_ORR_ (V)*** | | **d-band center** | | **Valence** | | **Spin states** | | **Spin Polarization** | **Refs** |
| --- | --- | --- | --- | --- | --- | --- | --- | --- | --- | --- | --- | --- | --- |
|  |  | **Before** | **After** | **Before** | **After** | **Before** | **After** | **Before** | **After** | **Before** | **After** |  |  |
| Built-in Electric Fields | coordination modification | MXene/FePc | Alk-MXene/FePc | 0.906 | 0.924 | − | − | 2.000 | 2.500 | − | − | yes | [73] |
|  | heterostructure | Fe−N−C | FeP@(N/P-C) | 0.721 | 0.822 | 1.010 | 1.510 | 3 | 3 | − | − | no | [74] |
|  |  | PtO | PdO@W_3_O_x_ | 0.690 | 0.890 | -2.670 | -2.570 | 2 | 2 | − | − | no | [75] |
|  |  | SMO_x_-MO | SMO_x_-SMO | 0.670 | 0.740 | -0.900 | -0.850 | 3.263 | 3.410 | − | − | no | [76] |
|  |  | Fe−N−C | Co_3_O_4_@Fe₁-NC | 0.900 | 0.927 | − | − | 0.700 | 1.200 | 1.794 | 1.877 | no | [77] |
|  |  | Fe_3_O_4_@N,O - CNSs | Fe_3_O_4_/La_2_O_3_@N,O - CNSs | 0.770 | 0.880 | -3.100 | -2.700 | 2.700 | 3.000 | − | − | no | [78] |
|  |  | CuCo_2_O_4_/NCNTs | MnCo_2_O_4_/NCNTs | 0.680 | 0.760 | -1.230 | -1.040 | 2.474 | 2.507 | − | − | no | [79] |
|  |  | CuCo_2_O_4_/NCNTs | CoCo_2_O_4_/NCNTs | 0.680 | 0.710 | -1.230 | -1.150 | 2.474 | 2.490 | − | − | no | [79] |
|  |  | CuCo_2_O_4_/NCNTs | NiCo_2_O_4_/NCNTs | 0.680 | 0.690 | -1.230 | -1.250 | 2.474 | 2.515 | − | − | no | [79] |
|  |  | CuCo_2_O_4_/NCNTs | ZnCo_2_O_4_/NCNTs | 0.680 | 0.710 | -1.230 | -1.580 | 2.474 | 2.598 | − | − | no | [79] |
| Crystal Fields | stress-strain | Pt | Pt-PLA | 0.850 | 0.910 | -3.240 | -3.440 | − | − | − | − | no | [80] |
|  |  | Pt | Pt-P1 | 0.850 | 0.940 | -3.240 | -3.520 | − | − | − | − | no | [79] |
|  | doping | Fe−N−C | Mn/Fe−N−C | 0.868 | 0.928 | − | − | − | − | 2.160 | 3.750 | no | [81] |
|  |  | Co−N−C | Mg_0.1_Co_0.9_−N−C | 0.800 | 0.860 | -1.318 | -1.341 | − | − | − | − | no | [82] |
|  |  | ZnIn_2_S_4_ | Ni_0.1_ZnIn_2_S_4_ | 0.721 | 0.806 | -1.720 | -1.758 | 2 | 2 | − | − | no | [83] |
|  |  | ZnIn_2_S_4_ | Ru_0.1_ZnIn_2_S_4_ | 0.721 | 0.845 | -1.720 | -1.827 | 2 | 3 | − | − | no | [83] |
|  | cluster | Fe−N−C | Fe-cluster@Fe−N−C | 0.770 | 0.860 | 0.000 | 0.000 | 2.586 | 2.538 | 2 | 1 | no | [84] |
|  |  | Fe−N−C | Pd_NC_@Fe−N−C | 0.820 | 0.870 | − | − | − | − |  |  | no | [85] |
|  |  | Fe−N−C | PtFe@Fe−N−C | 0.780 | 0.868 | − | − | − | − | 4.960 | 4.370 | no | [86] |
|  |  | Fe−N−C | Fe_3_@Fe−N−C | 0.898 | 0.936 | − | − | 2.720 | 2.800 | − | − | no | [87] |
|  |  | Fe−N−C | Fe_3_C@Fe−N−C | 0.860 | 0.895 | 1.115 | 1.06 | 3 | 3 | − | − | yes | [88] |
|  |  | Fe−N−C | Fe@Fe−N−C | 0.807 | 0.829 | -0.775 | -0.947 | − | − | − | − | no | [89] |
|  |  | Pt NWs | Pt@PtCu NW | 0.897 | 0.940 | -3.390 | -3.580 | 0.508 | 0.395 | − | − | no | [90] |
| Ligand Fields | defect/  vacancy | Fe−N−C | Fe SA/N_hc_C | 0.885 | 0.926 | -0.774 | -0.935 | 1.500 | 1.500 | − | − | no | [91] |
|  | stress-strain | S-Zn−N−C-850(p-Zn−N_4_−C) | S-Zn−N−C-950(b-Zn−N_4_−C) | 0.810 | 0.890 | -4.410 | -3.860 | 2 | 2 | − | − | no | [92] |
|  | heterostructure | PtSn/C | PtCo-PtSn/C | 0.884 | 0.930 | -2.260 | -2.450 | − | − | − | − | no | [93] |
|  | reconstruction | Pd/c-MnO_2_ | Pd/a-MnO_2_ | 0.830 | 0.870 | -5.630 | -6.190 | 4 | 4 | − | − | no | [94] |
|  | size | Ce−N−C-30 | Ce−N−C-50 | 0.680 | 0.780 | − | − | 3 | 3 | 1 | 3 | no | [95] |
|  |  | Pd Metallene | c-Pd M | 0.843 | 0.947 | -3.290 | -3.730 | − | − | − | − | no | [96] |
|  | doping | MoO_2_ | P-MoO_2_ | 0.720 | 0.780 | -2.150 | -1.630 | 4.000 | 3.500 | − | − | no | [97] |
|  |  | Cu SAC-700 | Cu SAC/P-700 | 0.830 | 0.870 | -3.410 | -3.330 | 2 | 2 | − | − | no | [98] |
|  |  | PFePc/C | PFePc/FC | 0.900 | 0.930 | -1.500 | -1.730 | 3.000 | 3.200 | − | − | no | [99] |
|  |  | Fe−N−C | Fe−B/N−C | 0.840 | 0.870 | -2.040 | -2.350 | 1.500 | 1.500 | − | − | no | [100] |
|  | coordination modification | CoN_4_/ZIF-8 | Meso-CoN_3_O/ZIF-8 | 0.830 | 0.910 | -2.580 | -2.310 | 2.350 | 2.180 | − | − | no | [101] |
|  |  | Fe–N_4_−C | Fe–N_4_−NPS-HC | 0.828 | 0.912 | − | − | 2.000 | 2.500 | 3.020 | 1.730 | yes | [102] |
|  |  | Fe−N_5_-LS | Fe−N_4_-HS | 0.867 | 0.916 | -1.536 | -1.698 | 2.500 | 1.000 | 2.160 | 6.080 | no | [103] |
|  |  | FeN_3_O | axial Fe−O−Ti coordination | 0.812 | 0.861 | -0.832 | -1.410 | 3 | 3 | 0 | 1 | no | [104] |
|  |  | Fe−N−C | FeN_3_S_1_-OH | 0.890 | 0.920 | -2.180 | -2.380 | 2.000 | 2.720 | − | − | no | [105] |
|  |  | Pt−N−C | Pt−N/P−C | 0.860 | 0.850 | -2.440 | -3.020 | − | − | − | − | no | [106] |
|  |  | Fe−N−C | Fe−N_3_S_1_−S | 0.870 | 0.930 | -1.204 | -1.785 | 2.000 | 2.300 | − | − | no | [107] |
|  |  | PNCH | FeNi-DSAs-PNCH | 0.740 | 0.890 | -0.380 | -1.030 | − | − | − | − | no | [108] |
|  |  | Fe−N−C | Fe-S/N-C | 0.878 | 0.920 | -3.400 | -4.530 | 2.500 | 2.500 | − | − | no | [109] |
|  |  | Zr−N_4_−C | O-Zr-N-C | 0.860 | 0.910 | -1.270 | -2.500 | 4.000 | 3.300 | − | − | no | [110] |
|  |  | Fe−N−C | B-doped Fe−N_4_−C | 0.834 | 0.859 | -1.300 | -1.100 | − | − | − | − | no | [111] |

*half-wave potential of ORR catalysts in 0.1 M KOH


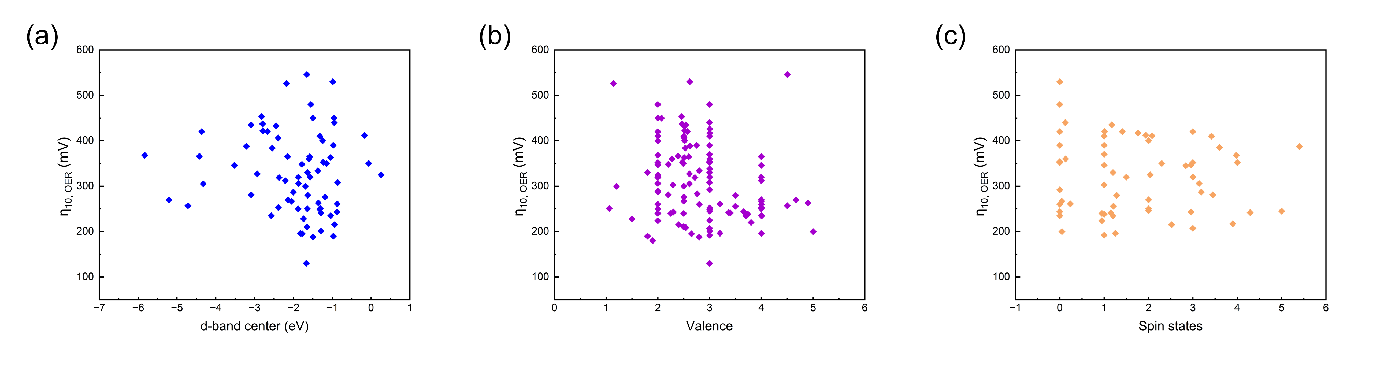


**Figure S1.** The distribution of OER overpotential with respect to different variable parameters: (a) d-band center, (b) valence, (c) spin state.


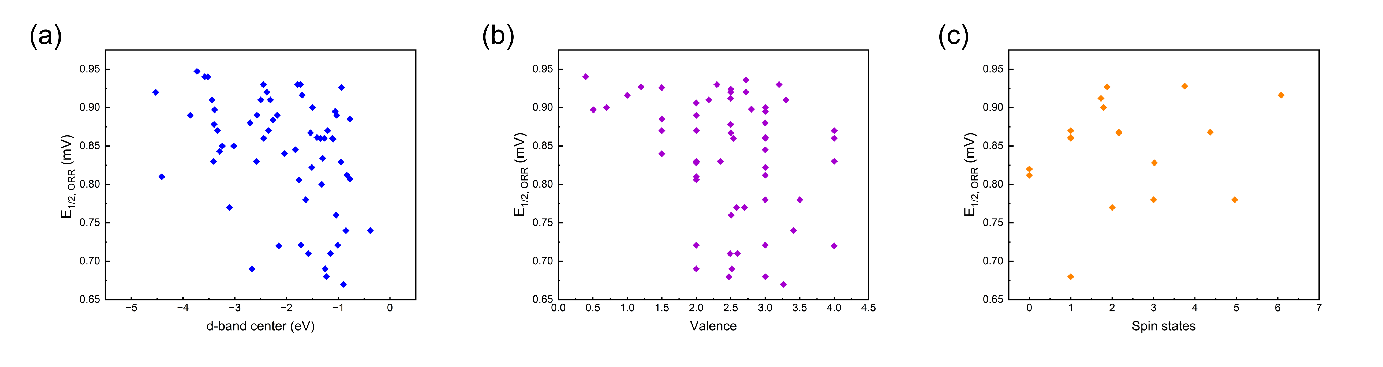


**Figure S2.** The distribution of ORR half-wave potential with respect to different variable parameters: (a) d-band center, (b) valence, (c) spin state.

1. **Machine Learning**

In our statistical analysis of ORR-related literature, instances with complete datasets encompassing d-band center, valence, and spin states were scarce, thereby limiting the feasibility of multivariate analysis. Consequently, machine learning was exclusively performed on OER samples with complete tri-parametric data to ensure statistical robustness in model training, yielding a covariance matrix heatmap of diverse variables (Figure 4b). The relevant code is provided below:

*import numpy as np*

*import pandas as pd*

*import seaborn as sns*

*import matplotlib.pyplot as plt*

*# read data*

*df = pd.read_excel('./spin-no-spin-OER.xlsx', skiprows=1, names = ['OER overpotential', 'd-band center', 'valence', 'spin states'])*

*df1 = df.iloc[:, 0:4]*

*corr_matrix = df1.corr()*

*# plot heatmap*

*font = {'family': 'serif',*

*'serif': 'Arial',*

*'weight': 'bold',*

*'size': 10}*

*plt.rc('font', **font)*

*plt.figure(figsize=(10, 6))*

*sns.heatmap(*

*corr_matrix,*

*annot=True,*

*cmap='RdYlGn_r',*

*linewidths=1,*

*vmin=-1,*

*vmax=1,*

*annot_kws={'size': 16, 'weight': 'bold'}*

*)*

*plt.xticks(rotation=0, fontsize=12, fontweight='bold')*

*plt.yticks(rotation=70, fontsize=12, fontweight='bold')*

*plt.savefig('./spin-no-spin_Covariance_Correlation_Matrix_Heatmap.png', dpi=500, bbox_inches='tight')*

# References

1. H. Singh, M. Marley-Hines, S. Chakravarty, M. Nath, Multi-walled carbon nanotube supported manganese selenide as a highly active bifunctional OER and ORR electrocatalyst, *J. Mater. Chem. A* **2022**, *10*, 6772.
2. M. Guo, X. Han, H. Feng, D.-F. Chai, W. Zhang, Y. Li, G. Dong, D. Guo, Spin-state-regulated dual-metal orbital engineering in heterostructured nanosheets for d-band center-optimized electrocatalytic water splitting, *J. Colloid Interface Sci.* **2025**, *696*, 137850.
3. S. Li, Y. Liu, K. Feng, C. Li, J. Xu, C. Lu, H. Lin, Y. Feng, D. Ma, J. Zhong, High Valence State Sites as Favorable Reductive Centers for High‐Current‐Density Water Splitting, *Angew. Chem. Int. Ed.* **2023**, *62, e202308670*.
4. X. Ren, T. Wu, Z. Gong, L. Pan, J. Meng, H. Yang, F. B. Dagbjartsdottir, A. Fisher, H.-J. Gao, Z. J. Xu, The origin of magnetization-caused increment in water oxidation, *Nat. Commun.* **2023**, 14, 2482.
5. Z. Xue, B. Wu, Z. Zhang, C. Lin, X. Li, Q. Zhang, K. Tao, Spin Selectivity Induced by the Interface Effect for Boosted Water Oxidation, *ACS Catal.* **2024**, 14, 5685.
6. C. Hao, Y. Wu, X. Zheng, Y. Du, Y. Fan, W. Pang, A. Tadich, S. Zhang, T. Frauenheim, T. Ma, X. Li, Z. Cheng, Engineering Magnetic Heterostructures with Synergistic Regulation of Charge‐Transfer and Spin‐Ordering for Enhanced Water Oxidation, *Adv. Sci.* **2025**, 12, 2409842.
7. L. Yang, R. He, M. Botifoll, Y. Zhang, Y. Ding, C. Di, C. He, Y. Xu, L. Balcells, J. Arbiol, Y. Zhou, A. Cabot, Enhanced Oxygen Evolution and Zinc‐Air Battery Performance via Electronic Spin Modulation in Heterostructured Catalysts, *Adv. Mater.* **2024**, 36, 2400572.
8. S. Zhou, X. Miao, X. Zhao, C. Ma, Y. Qiu, Z. Hu, J. Zhao, L. Shi, J. Zeng, Engineering electrocatalytic activity in nanosized perovskite cobaltite through surface spin-state transition, *Nat. Commun.* **2016**, 7, 11510.
9. G. Liu, F. Xie, X. Cai, J. Ye, Spin Crossover and Exchange Effects on Oxygen Evolution Reaction Catalyzed by Bimetallic Metal Organic Frameworks, *ACS Catal.* **2024**, 14, 8652.
10. J. Huang, C. N. Borca, T. Huthwelker, N. S. Yüzbasi, D. Baster, M. El Kazzi, C. W. Schneider, T. J. Schmidt, E. Fabbri, Surface oxidation/spin state determines oxygen evolution reaction activity of cobalt-based catalysts in acidic environment, *Nat. Commun.* **2024**, 15, 3067.
11. Z. Gao, J.-H. Liu, S. Wang, W. Yang, W. Wang, L. Li, H. Guo, J. Zheng, S. Ramakrishna, J. Zhang, L. Yang, Y.-Z. Long, Quenching controlled spin exchange interactions and spin selective electron transfer for oxygen evolution reactions, *Chem. Eng. J.* **2024**, *496*, 154216.
12. D. K. Cho, H. W. Lim, A. Haryanto, B. Yan, C. W. Lee, J. Y. Kim, Intercalation-Induced Irreversible Lattice Distortion in Layered Double Hydroxides, *ACS Nano* **2024**, *18*, 20459.
13. Y. Du, F. Xie, M. Lu, R. Lv, W. Liu, Y. Yan, S. Yan, Z. Zou, Continuous strain tuning of oxygen evolution catalysts with anisotropic thermal expansion, *Nat. Commun.* **2024**, 15, 1780.
14. W. H. Lee, M. H. Han, Y.-J. Ko, B. K. Min, K. H. Chae, H.-S. Oh, Electrode reconstruction strategy for oxygen evolution reaction: maintaining Fe-CoOOH phase with intermediate-spin state during electrolysis, *Nat. Commun.* **2022**, 13, 1780.
15. H. Xu, J. Qi, Y. Zhang, H. Liu, L. Hu, M. Feng, W. Lü, Magnetic Field-Enhanced Oxygen Evolution Reaction via the Tuneability of Spin Polarization in a Half-Metal Catalyst, *ACS Appl. Mater. Interfaces* **2023**, *15*, 32320.
16. G. Song, M. Wei, J. Zhou, L. Mu, S. Song, Modulation of the Phase Transformation of Fe_2_O_3_ for Enhanced Water Oxidation under a Magnetic Field, *ACS Catal.* **2024**, *14*, 846.
17. A. N. Nair, S. Fernandez, M. Marcos-Hernández, D. R. Romo, S. R. Singamaneni, D. Villagran, S. T. Sreenivasan, Spin-Selective Oxygen Evolution Reaction in Chiral Iron Oxide Nanoparticles: Synergistic Impact of Inherent Magnetic Moment and Chirality, *Nano Lett.* **2023**, *23*, 9042.
18. L. Lin, Y. Xu, Y. Han, R. Xu, T. Wang, Z. Sun, Z. Yan, Spin-Magnetic Effect of d-π Conjugation Polymer Enhanced O–H Cleavage in Water Oxidation, *J. Am. Chem. Soc.* **2024**, *146*, 7363.
19. L. Lin, R. Xin, M. Yuan, T. Wang, J. Li, Y. Xu, X. Xu, M. Li, Y. Du, J. Wang, S. Wang, F. Jiang, W. Wu, C. Lu, B. Huang, Z. Sun, J. Liu, J. He, G. Sun, Revealing Spin Magnetic Effect of Iron-Group Layered Double Hydroxides with Enhanced Oxygen Catalysis, *ACS Catal.* **2023**, 13, 1431.
20. X. Bai, S. Lu, P. Song, Z. Jia, Z. Gao, T. Peng, Z. Wang, Q. Jiang, H. Cui, W. Tian, R. Feng, Z. Liang, Q. Kang, H. Yuan, Heterojunction of MXenes and MN4–graphene: Machine learning to accelerate the design of bifunctional oxygen electrocatalysts, *J. Colloid Interface Sci.* **2024**, *664*, 716.
21. A. Vadakkayil, C. Clever, K. N. Kunzler, S. Tan, B. P. Bloom, D. H. Waldeck, Chiral electrocatalysts eclipse water splitting metrics through spin control, *Nat. Commun.* **2023**, 14, 1067.
22. Z. Jia, Y. Yuan, Y. Zhang, X. Lyu, C. Liu, X. Yang, Z. Bai, H. Wang, L. Yang, Optimizing 3d spin polarization of CoOOH by in situ Mo doping for efficient oxygen evolution reaction, *Carbon Energy* **2024**, 6, e418.
23. T. Liu, H. Guo, Q. Zhang, M. Fujishige, M. Endo, Z. Zhang, F. Wang, Insulator‐Transition‐Induced Degradation of Pyrochlore Ruthenates in Electrocatalytic Oxygen Evolution and Stabilization through Doping, *Angew. Chem. Int. Ed.* **2024**, *63,* e202412139.
24. J. Zhang, Y. Zhao, W. Zhao, J. Wang, Y. Hu, C. Huang, X. Zou, Y. Liu, D. Zhang, X. Lu, H. Fan, Y. Hou, Improving Electrocatalytic Oxygen Evolution through Local Field Distortion in Mg/Fe Dual‐site Catalysts, *Angew. Chem. Int. Ed.* **2023**, 62, e202314303.
25. L. Li, J. Zhou, X. Wang, J. Gracia, M. Valvidares, J. Ke, M. Fang, C. Shen, J.-M. Chen, Y.-C. Chang, C.-W. Pao, S.-Y. Hsu, J.-F. Lee, A. Ruotolo, Y. Chin, Z. Hu, X. Huang, Q. Shao, Spin‐Polarization Strategy for Enhanced Acidic Oxygen Evolution Activity, *Adv. Mater.* **2023**, *35*, 2302966.
26. H. Li, Q. Quan, H. Dong, Y. Zhang, P. Xie, D. Chen, D. Yin, C. Wong, J. C. Ho, Hierarchical Spin‐Polarized Nanosheet Array for Boosting Ampere‐Level Water Oxidation Under Magnetic Field, *Adv. Funct. Mater.* **2025**, *35*, 2420810.
27. J.-Y. Zhang, Y. Yan, B. Mei, R. Qi, T. He, Z. Wang, W. Fang, S. Zaman, Y. Su, S. Ding, B. Y. Xia, Local spin-state tuning of cobalt–iron selenide nanoframes for the boosted oxygen evolution, *Energy Environ. Sci.* **2021**, *14*, 365.
28. Z. Wang, S. Shen, Z. Lin, W. Tao, Q. Zhang, F. Meng, L. Gu, W. Zhong, Regulating the Local Spin State and Band Structure in Ni_3_S_2_ Nanosheet for Improved Oxygen Evolution Activity, *Adv. Funct. Mater.* **2022**, 32, 2112832.
29. L. Tan, X. Wu, H. Wang, J. Zeng, B. Mei, X. Pan, W. Hu, M. Faiza, Q. Xiao, Y. Zhao, C. Fu, C. Lin, X. Li, W. Luo, Ferromagnetic-Interaction-Induced Spin Symmetry Broken in Ruthenium Oxide for Enhanced Acidic Water Oxidation, *ACS Catal.* **2024**, *14*, 11273.
30. J. Ran, L. Wang, M. Si, X. Liang, D. Gao, Tailoring Spin State of Perovskite Oxides by Fluorine Atom Doping for Efficient Oxygen Electrocatalysis, *Small* **2023***, 19*, 2206367.
31. Y. Liu, L. Li, X. Li, Y. Xu, D. Wu, T. Sakthivel, Z. Guo, X. Zhao, Z. Dai, Asymmetric tacticity navigates the localized metal spin state for sustainable alkaline/sea water oxidation, *Sci. Adv.* **2025**, *11*, eads0861.
32. J. Liu, Z. Yu, J. Huang, S. Yao, R. Jiang, Y. Hou, W. Tang, P. Sun, H. Huang, M. Wang, Redox-active ligands enhance oxygen evolution reaction activity: Regulating the spin state of ferric ions and accelerating electron transfer, *J. Colloid Interface Sci.* **2023**, *650*, 1182.
33. H. Jia, N. Yao, Z. Liao, L. Wu, J. Zhu, Y. Lao, W. Luo, Understanding the Role of Spin State in Cobalt Oxyhydroxides for Water Oxidation, *Angew. Chem. Int. Ed.* **2024**, *63,* e202408005.
34. Z.-D. He, R. Tesch, M. J. Eslamibidgoli, M. H. Eikerling, P. M. Kowalski, Low-spin state of Fe in Fe-doped NiOOH electrocatalysts, *Nat. Commun.* **2023**, *14*, 3498.
35. Z.-J. Zhang, J.-P. Guo, S.-H. Sun, Q. Sun, Y.-W. Zhao, Y.-F. Zhang, Z.-Y. Yu, C.-S. Li, Y. Sun, M.-M. Zhang, Y. Jiang, Optimized valence state of Co and Ni in high-entropy alloy for high active-stable OER, *Rare Met.* **2023**, *42*, 3607.
36. Z. Wang, J. Huang, L. Wang, Y. Liu, W. Liu, S. Zhao, Z. Liu, Cation‐Tuning Induced d‐Band Center Modulation on Co‐Based Spinel Oxide for Oxygen Reduction/Evolution Reaction, *Angew. Chem. Int. Ed.* **2022**, *61*, e202114696.
37. W. Cao, X.-H. Gao, J. Wu, A.-Q. Huang, H. Hu, Z.-W. Chen, Regulating the Spin Polarization of NiFe Layered Double Hydroxide for the Enhanced Oxygen Evolution Reaction, *ACS Catal.* **2024**, *14*, 3640.
38. F. He, Q. Zheng, X. Yang, L. Wang, Z. Zhao, Y. Xu, L. Hu, Y. Kuang, B. Yang, Z. Li, L. Lei, M. Qiu, J. Lu, Y. Hou, Spin‐State Modulation on Metal–Organic Frameworks for Electrocatalytic Oxygen Evolution, *Adv. Mater.* **2023**, *35*, 2304022.
39. J. Guo, G. Wang, S. Cui, B. Xia, Z. Liu, S. Zang, Vacancy and strain engineering of Co3O4 for efficient water oxidation, *J. Colloid Interface Sci.* **2023**, *629*, 346.
40. X. Zhang, R. Xu, T. Wang, L. Niu, Y. Gong, C. Li, Enhancing electrocatalytic performance in the oxygen evolution reaction of zirconium-based amorphous high-entropy oxides via controlled introduction of oxygen vacancies: experimental insights and DFT simulations, *J. Colloid Interface Sci.* **2025**, *694*, 137635.
41. X. Gao, H. Liu, Y. Wang, J. Guo, X. Sun, W. Sun, H. Zhao, J. Bai, C. Li, Tailoring the d-band electronic structure of deficient LaMn0.3Co0.7O3-δ perovskite nanofibers for boosting oxygen electrocatalysis in Zn-Air batteries, *J. Colloid Interface Sci.* **2023**, *650*, 951.
42. J. Ran, M. Si, D. Gao, Co@CoO chiral nanostructures enabling efficient oxygen electrocatalysis by modulated spin-polarization, *Chem. Eng. J.* **2024**, *493*, 152545.
43. Z. Du, Z. Meng, X. Gong, Z. Hao, X. Li, H. Sun, X. Hu, S. Yu, H. Tian, Rapid Surface Reconstruction of Pentlandite by High‐Spin State Iron for Efficient Oxygen Evolution Reaction, *Angew. Chem. Int. Ed.* **2024**, *63*, e202317022.
44. X. Wang, W. Zhou, S. Zhai, X. Chen, Z. Peng, Z. Liu, W. Deng, H. Wu, Metal‐Organic Frameworks: Direct Synthesis by Organic Acid‐Etching and Reconstruction Disclosure as Oxygen Evolution Electrocatalysts, *Angew. Chem. Int. Ed.* **2024**, *63*, e202400323.
45. Z. Zhang, J. Sun, J. Zhao, M. Wu, R. Liu, Enhanced electrocatalytic water splitting by manipulating d-band center of aligned MoXS (X = Co, Ni, Fe) heteronanosheet, *Appl. Surf. Sci* **2023**, *640*, 158329.
46. X. Li, Y. Liu, C. Li, H. Xue, S. Chen, Q. Xu, H. Pang, Tuning the Electronic Property of Reconstructed Atomic Ni‐CuO Cluster Supported on N/O‐C for Electrocatalytic Oxygen Evolution, *Adv. Sci.* **2024**, *11*.
47. J. Zhu, X. Peng, P. Xi, C. Jia, D. Gao, Dual-Channel Regulation of Spin Polarization Achieves 1 + 1 > 2 Electrocatalytic Performance in Spinel Ferrites, *Nano Lett.* **2025**, *25*, 10337.
48. A. Vadakkayil, W. A. Dunlap-Shohl, M. Joy, B. P. Bloom, D. H. Waldeck, Improved Catalyst Performance for the Oxygen Evolution Reaction under a Chiral Bias, *ACS Catal.* **2024**, *14*, 17303.
49. J. Zhang, Y. Zhao, W. Zhao, J. Wang, Y. Hu, C. Huang, X. Zou, Y. Liu, D. Zhang, X. Lu, H. Fan, Y. Hou, Improving Electrocatalytic Oxygen Evolution through Local Field Distortion in Mg/Fe Dual‐site Catalysts, *Angew. Chem. Int. Ed.* **2023**, *62*.
50. R. R. Chen, Y. Sun, S. J. H. Ong, S. Xi, Y. Du, C. Liu, O. Lev, Z. J. Xu, Antiferromagnetic Inverse Spinel Oxide LiCoVO_4_ with Spin‐Polarized Channels for Water Oxidation, *Adv. Mater.* **2020**, *32*, 1907976.
51. Z. Lv, Z. Shu, J. Luo, J. Xu, Y. Ma, L. Zhang, H. Xu, Z. Mao, Asymmetric high-coordination Co-NSP single-atom catalysts with tailored d-p-orbital electron structure for efficient bifunctional catalyst of rechargeable Zn-air battery cathodes, *ACB-Env.* **2025**, *365*, 124889.
52. W. Liu, D. Zheng, T. Deng, Q. Chen, C. Zhu, C. Pei, H. Li, F. Wu, W. Shi, S. Yang, Y. Zhu, X. Cao, Boosting Electrocatalytic Activity of 3d‐Block Metal (Hydro)oxides by Ligand‐Induced Conversion, *Angew. Chem. Int. Ed.* **2021**, *60*, 10614.
53. J. Zhou, Z. Han, X. Wang, H. Gai, Z. Chen, T. Guo, X. Hou, L. Xu, X. Hu, M. Huang, S. V. Levchenko, H. Jiang, Discovery of Quantitative Electronic Structure‐OER Activity Relationship in Metal‐Organic Framework Electrocatalysts Using an Integrated Theoretical‐Experimental Approach, *Adv. Funct. Mater.* **2021**, *31*, 2102066.
54. M. Cai, Q. Zhu, X. Wang, Z. Shao, L. Yao, H. Zeng, X. Wu, J. Chen, K. Huang, S. Feng, Formation and Stabilization of NiOOH by Introducing α‐FeOOH in LDH: Composite Electrocatalyst for Oxygen Evolution and Urea Oxidation Reactions, *Adv. Mater.* **2023**, *35*, 2209338.
55. C. Liang, H. Ai, L. Lin, X. Lu, L. Li, H. Zhang, P. Wang, Z. Zheng, Z. Wang, H. Cheng, Y. Dai, D. Xing, B. Huang, Y. Liu, The Cu─O─Co Asymmetric Bimetallic Sites Constructed by Ion‐Exchange for Efficient Oxygen Evolution Reaction, *Small* **2025**, *21*, 2500744.
56. C. Zhao, J. Wang, Y. Gao, J. Zhang, C. Huang, Q. Shi, S. Mu, Q. Xiao, S. Huo, Z. Xia, J. Zhang, X. Lu, Y. Zhao, D‐Orbital Manipulated Ru Nanoclusters for High‐Efficiency Overall Water Splitting at Industrial‐Level Current Densities, *Adv. Funct. Mater.* **2024**, *34*, 2307917.
57. W. S. Lee, H. Maeda, Y. Kuo, K. Muraoka, N. Fukui, K. Takada, S. Sasaki, H. Masunaga, A. Nakayama, H. Tian, H. Nishihara, K. Sakaushi, Spontaneous‐Spin‐Polarized 2D π‐d Conjugated Frameworks Towards Enhanced Oxygen Evolution Kinetics, *Small* **2024**, 20, 2401987.
58. L. Li, Y. Wang, R. R. Nazmutdinov, R. R. Zairov, Q. Shao, J. Lu, Magnetic Field Enhanced Cobalt Iridium Alloy Catalyst for Acidic Oxygen Evolution Reaction, *Nano Lett.* **2024**, *24*, 6148.
59. L. Hu, F. Wang, Y. Jing, High Catalytic Activity of Co-centered 2D Metal Organic Frameworks toward Bifunctional Oxygen Evolution and Reduction Reactions: Rationalized by Spin Polarization Effect, *J. Phys. Chem. Lett.* **2023**, *14*, 11429.
60. U. Utkarsh, Chiral supramolecular polymer functionalized two-dimensional transition metal-based catalyst for enhancing the electrochemical water splitting via spin-polarized charge transfer, *J. Mater. Chem. A*, **2024**, 12, 20354–20363.
61. X. Mu, K. Wang, K. Lv, B. Feng, X. Yu, L. Li, X. Zhang, X. Yang, Z. Lu, Doping of Cr to Regulate the Valence State of Cu and Co Contributes to Efficient Water Splitting, *ACS Appl. Mater. Interfaces* **2023**, *15*, 16552.
62. P. Zhang, S. Liu, J. Zhou, L. Zhou, B. Li, S. Li, X. Wu, Y. Chen, X. Li, X. Sheng, Y. Liu, J. Jiang, Co‐Adjusting d‐Band Center of Fe to Accelerate Proton Coupling for Efficient Oxygen Electrocatalysis, *Small* **2024**, *20*, 2307662.
63. T. Yu, R. Ding, F. Quan, Z. Ni, X. Liu, Y. He, W. Mu, X. Lei, Y. Mo, Ag improves the performance of the oxygen evolution reaction by lowering the D-band center of the active site Ni, *Int. J. Hydrogen Energy* **2024**, *51*, 935.
64. S.-Y. Lu, T. Hu, C. Wu, J. He, J. Zhang, R. Wang, Y. Liu, M. Jin, Breaking the symmetry and d-orbital optimization at Co site in CoNC as bifunctional air catalysts for rechargeable liquid and flexible solid-state Zn-air batteries, *J. Colloid Interface Sci.* **2025**, *693*, 137588.
65. X. Cui, Y. Tao, X. Xu, G. Yang, P-doping optimized d-band center position in MoO2 with enhanced oxygen reduction reaction and oxygen evolution reaction activities for rechargeable Zn-air battery, *J. Power Sources* **2023**, *557*, 232519.
66. X. Wu, Z. Shao, Q. Zhu, X. Hou, C. Wang, J. Zeng, K. Huang, S. Feng, Tuning the d-Band Center of Co_3_O_4_ via Octahedral and Tetrahedral Codoping for Oxygen Evolution Reaction, *ACS Catal.* **2024**, *14*, 5888.
67. L. Zhang, W. Li, S. Ren, W. Song, C. Wang, X. Lu, Manipulating *d*‐Band Center of Ru Sites in Branched RuO_2_ Nanofibers Enables Significantly Enhanced Alkaline Overall Water Splitting Performance, *Adv. Energy Mater.* **2025**, *15*.
68. Z. Ji, Y. Jin, K. Tang, B. Li, J. Sun, X. Zhu, X. Yao, Z. Liu, X. Lang, Y. Zhu, Q. Jiang, Precisely manipulating the d-band center of Ni1−xCoxMoO4·nH2O for enhanced alkaline oxygen evolution with low overpotential, *J. Colloid Interface Sci.* **2025**, *698*, 138119.
69. J. Zhou, P. Li, X. Xia, Y. Zhao, Z. Hu, Y. Xie, L. Yang, Y. Liu, Y. Du, Q. Zhou, L. Yu, Y. Yu, Precisely tailoring the d-band center of nickel sulfide for boosting overall water splitting, *ACB-Env.* **2024**, *359*, 124461.
70. J.-Y. Xie, F.-L. Wang, X.-J. Zhai, X. Li, Y.-S. Zhang, R.-Y. Fan, R.-Q. Lv, Y.-M. Chai, B. Dong, Manganese doped hollow cobalt oxide catalysts for highly efficient oxygen evolution in wide pH range, *Chem. Eng. J.* **2024**, *482*, 148926.
71. Y. Huang, M. Li, F. Pan, Z. Zhu, H. Sun, Y. Tang, G. Fu, Plasma‐induced Mo‐doped Co_3_O_4_ with enriched oxygen vacancies for electrocatalytic oxygen evolution in water splitting, *Carbon Energy* **2023**, *5*.
72. S. Xu, D. Jiao, X. Ruan, Z. Jin, Y. Qiu, J. Fan, L. Zhang, W. Zheng, X. Cui, Synergistic modulation of the d-band center in Ni_3_S_2_ by selenium and iron for enhanced oxygen evolution reaction (OER) and urea oxidation reaction (UOR), *J. Colloid Interface Sci.* **2024**, *671*, 46.
73. Y. Dai, B. Liu, Z. Zhang, P. Guo, C. Liu, Y. Zhang, L. Zhao, Z. Wang, Tailoring the d‐Orbital Splitting Manner of Single Atomic Sites for Enhanced Oxygen Reduction, *Adv. Mater.* **2023**, *35*.
74. L.-H. Xu, P.-C. Che, X.-J. Zhang, S. Cosnier, D. Shan, FeP nanoparticles highly dispersed on N,P-doped petaloid carbon nanosheet: Interface engineering and boosted intrinsic ORR activity, *Appl. Surf. Sci* **2023**, *620*, 156770.
75. J.-H. Lee, D. Yim, J. H. Park, C. H. Lee, J.-M. Ju, S. U. Lee, J.-H. Kim, Tuning d-band centers by coupling PdO nanoclusters to WO_3_ nanosheets to promote the oxygen reduction reaction, *J. Mater. Chem. A* **2020**, *8*, 13490.
76. C. Chen, X. Wang, J. Zhong, J. Liu, G. I. N. Waterhouse, Z. Liu, Epitaxially Grown Heterostructured SrMn_3_O_6−_*_x_*‐SrMnO_3_ with High‐Valence Mn^3+/4+^ for Improved Oxygen Reduction Catalysis, *Angew. Chem. Int. Ed.* **2021**, *60*, 22043.
77. Y. Xie, Y. Feng, S. Zhu, Y. Yu, H. Bao, Q. Liu, F. Luo, Z. Yang, Modulation in Spin State of Co_3_O_4_ Decorated Fe Single Atom Enables a Superior Rechargeable Zinc‐Air Battery Performance, *Adv. Mater.* **2025**, *37*.
78. T. Li, L. Zhang, L. Zhang, J. Ke, T. Du, L. Zhang, Y. Cao, C. Yan, T. Qian, Tailoring the Chemisorption Manner of Fe d‐Band Center with La_2_O_3_ for Enhanced Oxygen Reduction in Anion Exchange Membrane Fuel Cells, *Adv. Funct. Mater.* **2024**, *34*.
79. Z. Wang, J. Huang, L. Wang, Y. Liu, W. Liu, S. Zhao, Z. Liu, Cation‐Tuning Induced d‐Band Center Modulation on Co‐Based Spinel Oxide for Oxygen Reduction/Evolution Reaction, *Angew. Chem. Int. Ed.* **2022**, *61*.
80. Q. Hua, X. Chen, J. Chen, N. M. Alghoraibi, Y. Lee, T. J. Woods, R. T. Haasch, S. C. Zimmerman, A. A. Gewirth, Inducing Microstrain in Electrodeposited Pt through Polymer Addition for Highly Active Oxygen Reduction Catalysis, *ACS Catal.* **2024**, *14*, 7526.
81. G. Yang, J. Zhu, P. Yuan, Y. Hu, G. Qu, B.-A. Lu, X. Xue, H. Yin, W. Cheng, J. Cheng, W. Xu, J. Li, J. Hu, S. Mu, J.-N. Zhang, Regulating Fe-spin state by atomically dispersed Mn-N in Fe-N-C catalysts with high oxygen reduction activity, *Nat. Commun.* **2021**, *12*.
82. H. Wang, X. Niu, W. Liu, R. Yin, J. Dai, W. Guo, C. Kong, L. Ma, X. Ding, F. Wu, W. Shi, T. Deng, X. Cao, S‐Block Metal Mg‐Mediated Co─N─C as Efficient Oxygen Electrocatalyst for Durable and Temperature‐Adapted Zn–Air Batteries, *Adv. Sci.* **2024**.
83. Z. Hou, Z. Sun, C. Cui, D. Zhu, Y. Yang, T. Zhang, Ru Coordinated ZnIn_2_S_4_ Triggers Local Lattice‐Strain Engineering to Endow High‐Efficiency Electrocatalyst for Advanced Zn‐Air Batteries, *Adv. Funct. Mater.* **2022**, *32*.
84. C. Chen, Y. Wu, X. Li, Y. Ye, Z. Li, Y. Zhou, J. Chen, M. Yang, F. Xie, Y. Jin, C. Jones, N. Wang, H. Meng, S. Chen, Modulating Fe spin state in FeNC catalysts by adjacent Fe atomic clusters to facilitate oxygen reduction reaction in proton exchange membrane fuel cell, *ACB-Env.* **2024**, *342*, 123407.
85. X. Wei, S. Song, W. Cai, X. Luo, L. Jiao, Q. Fang, X. Wang, N. Wu, Z. Luo, H. Wang, Z. Zhu, J. Li, L. Zheng, W. Gu, W. Song, S. Guo, C. Zhu, Tuning the spin state of Fe single atoms by Pd nanoclusters enables robust oxygen reduction with dissociative pathway, *Chem* **2023**, *9*, 181.
86. D. Xue, Y. Yuan, Y. Yu, S. Xu, Y. Wei, J. Zhang, H. Guo, M. Shao, J.-N. Zhang, Spin occupancy regulation of the Pt d-orbital for a robust low-Pt catalyst towards oxygen reduction, *Nat. Commun.* **2024**, *15*, 5990.
87. G. Yang, H. Cai, Z. Xu, C. Ji, Z. Yang, S. Zhang, Y. Zhang, B. Wang, B. Mei, C. Liang, S. Yang, Spin polarization regulation of Fe–N4 by Fe3 atomic clusters for highly active oxygen reduction reaction, *Sci. Bull.* **2025**, *70*, 1793.
88. G. Li, J. Liu, C. Xu, H. Chen, H. Hu, R. Jin, L. Sun, H. Chen, C. Guo, H. Li, Y. Si, Regulating the Fe-spin state by Fe/Fe3C neighbored single Fe-N4 sites in defective carbon promotes the oxygen reduction activity, *Energy Storage Materials* **2023**, *56*, 394.
89. L. Li, Y. Wen, G. Han, F. Kong, L. Du, Y. Ma, P. Zuo, C. Du, G. Yin, Architecting FeN*_x_* on High Graphitization Carbon for High‐Performance Oxygen Reduction by Regulating d‐Band Center, *Small* **2023**, *19*, 2300758.
90. J. Luo, S. Zhang, F. Liu, H. Cui, X. Liu, H. Liao, Y. Gu, M. Liu, P. Tan, J. Pan, Manipulating d‐band Center by Interface‐Induced Dislocation in Pt@PtCu Nanowires Boosting Oxygen Reduction, *Adv. Funct. Mater.* **2025**, *35*, 2422533.
91. Q. Wang, L. Lyu, X. Hu, W. Fan, C. Shang, Q. Huang, Z. Li, Z. Zhou, Y. Kang, Tailoring the Surface Curvature of the Supporting Carbon to Tune the d‐Band Center of Fe−N−C Single‐Atom Catalysts for Zinc‐Urea‐Air Batteries, *Angew. Chem. Int. Ed.* **2025**, *64*, e202422920.
92. Y. Tan, Z. Zhang, S. Chen, W. Wu, L. Yu, R. Chen, F. Guo, Z. Wang, N. Cheng, Local Geometric Distortion to Stimulate Oxygen Reduction Activity of Atomically Dispersed Zn‐N_x_ Sites for Zn–Air Batteries, *Adv. Funct. Mater.* **2024**, *34*, 2311337.
93. J. Chen, G. Qian, B. Chu, Z. Jiang, K. Tan, L. Luo, B. Li, S. Yin, Tuning d‐Band Center of Pt by PtCo‐PtSn Heterostructure for Enhanced Oxygen Reduction Reaction Performance, *Small* **2022**, *18*, 2106773.
94. Y. Wang, J. Liu, H. Yuan, F. Liu, T. Hu, B. Yang, Strong Electronic Interaction between Amorphous MnO_2_ Nanosheets and Ultrafine Pd Nanoparticles toward Enhanced Oxygen Reduction and Ethylene Glycol Oxidation Reactions, *Adv. Funct. Mater.* **2023**, *33*, 2211909.
95. Y. Zhao, H. Wang, J. Li, Y. Fang, Y. Kang, T. Zhao, C. Zhao, Regulating the Spin‐State of Rare‐Earth Ce Single Atom Catalyst for Boosted Oxygen Reduction in Neutral Medium, *Adv. Funct. Mater.* **2023**, *33*, 2305268.
96. F. Lin, F. Lv, Q. Zhang, H. Luo, K. Wang, J. Zhou, W. Zhang, W. Zhang, D. Wang, L. Gu, S. Guo, Local Coordination Regulation through Tuning Atomic‐Scale Cavities of Pd Metallene toward Efficient Oxygen Reduction Electrocatalysis, *Adv. Mater.* **2022**, *34*, 2202084.
97. X. Cui, Y. Tao, X. Xu, G. Yang, P-doping optimized d-band center position in MoO2 with enhanced oxygen reduction reaction and oxygen evolution reaction activities for rechargeable Zn-air battery, *J. Power Sources* **2023**, *557*, 232519.
98. L. Wang, W.-W. Tian, W. Zhang, F. Yu, Z.-Y. Yuan, Boosting oxygen electrocatalytic performance of Cu atom by engineering the d-band center via secondary heteroatomic phosphorus modulation, *Appl. Catal. B* **2023**, *338*, 123043.
99. K.-Z. Yang, Y.-Z. Su, C. Xu, P.-P. Guo, Y.-M. Zhao, X. Liu, L. Jia, Y. Yang, H.-N. Zhu, P.-J. Wei, J.-G. Liu, Boosting Oxygen Reduction Reaction through Substrate Fluorination-Mediated d-Band Center Tuning and Microenvironment Optimization of Molecular Catalysts, *ACS Catal.* **2025**, *15*, 8114.
100. W. Zhang, S. Zhang, P. Guo, H. Chen, Y. Zhou, F. Yu, Efficient and durable oxygen reduction in alkaline media by doping heteroatomic boron into FeSA-NC catalyst, *J. Colloid Interface Sci.* **2024**, *669*, 896.
101. X. Wang, S. Zhang, M. Li, Y. Wan, Z. Sun, R. Li, Z. Zhu, H. Wu, Z. Zhao, S. Hu, F. Bu, D. Chao, W. Luo, Tailored Design of Mesoporous Metal Organic Framework Single Crystals by Kinetics‐Mediated Micelle Assembly for Efficient Asymmetrical Single‐Atom Catalysis, *Adv. Mater.* **2025**, *37*, 2500370.
102. J. Liu, W. Chen, S. Yuan, T. Liu, Q. Wang, High-coordination Fe–N_4_SP single-atom catalysts *via* the multi-shell synergistic effect for the enhanced oxygen reduction reaction of rechargeable Zn–air battery cathodes, *Energy Environ. Sci.* **2024**, *17*, 249.
103. D. Xue, P. Yuan, S. Jiang, Y. Wei, Y. Zhou, C.-L. Dong, W. Yan, S. Mu, J.-N. Zhang, Altering the spin state of Fe-N-C through ligand field modulation of single-atom sites boosts the oxygen reduction reaction, *Nano Energy* **2023**, *105*, 108020.
104. Y. Liu, X. Liu, Z. Lv, R. Liu, L. Li, J. Wang, W. Yang, X. Jiang, X. Feng, B. Wang, Tuning the Spin State of the Iron Center by Bridge‐Bonded Fe‐O‐Ti Ligands for Enhanced Oxygen Reduction, *Angew. Chem. Int. Ed.* **2022**, *61*.
105. S. Zhou, C. Chen, J. Xia, L. Li, X. Qian, F. Yin, G. He, Q. Chen, H. Chen, FeN_3_S_1_─OH Single‐Atom Sites Anchored on Hollow Porous Carbon for Highly Efficient pH‐Universal Oxygen Reduction Reaction, *Small* **2024**, *20*.
106. X. Zhu, X. Tan, K. Wu, S. Haw, C. Pao, B. Su, J. Jiang, S. C. Smith, J. Chen, R. Amal, X. Lu, Intrinsic ORR Activity Enhancement of Pt Atomic Sites by Engineering the *d*‐Band Center via Local Coordination Tuning, *Angew. Chem. Int. Ed.* **2021**, *60*, 21911.
107. K. Chi, Z. Wang, T. Sun, P. He, F. Xiao, J. Lu, S. Wang, Simultaneously Engineering the First and Second Coordination Shells of Single Iron Catalysts for Enhanced Oxygen Reduction, *Small* **2024**, *20*.
108. B. Wang, J. Tang, X. Zhang, M. Hong, H. Yang, X. Guo, S. Xue, C. Du, Z. Liu, J. Chen, Nitrogen doped porous carbon polyhedral supported Fe and Ni dual-metal single-atomic catalysts: template-free and metal ligand-free synthesis with microwave-assistance and d-band center modulating for boosted ORR catalysis in zinc-air batteries, *Chem. Eng. J.* **2022**, *437*, 135295.
109. H. Liu, C. Wang, C. Liu, X. Zong, Y. Wang, X. Huang, Z. Hu, Z. Zhang, Coordination Engineering Induced d-Band Center Shift on Single-Atom Fe Electrocatalysts for Enhanced Oxygen Reduction, *ACS Appl. Mater. Interfaces* **2023**, *15*, 29110.
110. X. Wang, Y. An, L. Liu, L. Fang, Y. Liu, J. Zhang, H. Qi, T. Heine, T. Li, A. Kuc, M. Yu, X. Feng, Atomically Dispersed Pentacoordinated‐Zirconium Catalyst with Axial Oxygen Ligand for Oxygen Reduction Reaction, *Angew. Chem. Int. Ed.* **2022**, *134*, e202209746.
111. X. Li, J. Liu, Q. Cai, Z. Kan, S. Liu, J. Zhao, Engineering d-band center of iron single atom site through boron incorporation to trigger the efficient bifunctional oxygen electrocatalysis, *J. Colloid Interface Sci.* **2022**, *628*, 331.
